# Direct observations of submesoscale turbulence driving mesoscale eddy leakage

**DOI:** 10.1126/sciadv.aef8067

**Published:** 2026-10-23

**Authors:** Haijin Cao, Zhiyou Jing, Baylor Fox-Kemper, Bo Qiu, Joseph H. Lacasce, Tong Yan

**Affiliations:** ^1^ Key Laboratory of Marine Hazards Forecasting, Ministry of Natural Resources, Hohai University, Nanjing, China.; ^2^ College of Oceanography, Hohai University, Nanjing, China.; ^3^ State Key Laboratory of Tropical Oceanography, South China Sea Institute of Oceanology, Chinese Academy of Sciences, Guangzhou, China.; ^4^ Department of Earth, Environmental, and Planetary Sciences, Brown University, Providence, RI, USA.; ^5^ Department of Oceanography, University of Hawaii at Manoa, Honolulu, HI, USA.; ^6^ Department of Geosciences, University of Oslo, Oslo, Norway.

## Abstract

Understanding oceanic transport and material fate has long relied on observations that primarily resolve mesoscale, largely geostrophic flows, leaving the role of submesoscale turbulence comparatively unobserved and poorly understood. Here, using new high-resolution drifter-cluster experiments with closest initial spacings below 1 km, we provide direct Lagrangian observations of how submesoscale turbulence modifies mesoscale eddy coherence and transport pathways. The clustered drifters resolve intense, transient ageostrophic motions that are fundamentally absent from geostrophic descriptions of eddy behavior. Analyzed in an eddy-centered reference frame, the observations reveal pronounced submesoscale-driven radial dispersion that facilitates substantial water-mass exchange between the eddy core and its periphery in both the mixed layer and the subsurface. Quantitative estimates indicate submesoscale horizontal diffusivity of *O*(10 to 100) m^2^/s and a nonlocal dispersion regime across submesoscales. These results challenge the traditional view of mesoscale eddies as coherent structures containing a persistent water mass, with broad implications for interpreting and predicting the transport and fate of tracers in the ocean.

## INTRODUCTION

The transport and redistribution of heat, salt, nutrients, carbon, pollutants, and other tracers in the ocean arise from the interaction among dynamical processes spanning basin-scale circulation, mesoscale eddies, and submesoscale turbulence. Together, these multiscale processes shape the ocean’s stratification, regulate biogeochemical pathways, and ultimately influence Earth’s climate system and marine ecosystems (*1*–*4*). However, current observational assessments of ocean transport rely largely on low-resolution satellite altimetry, which primarily resolves mesoscale, geostrophic motions. Consequently, mesoscale eddies are commonly interpreted as coherent vortices capable of trapping and transporting water masses over long distances (*5*, *6*). This paradigm treats mesoscale eddies as materially closed structures, with pronounced Lagrangian coherent structures (LCSs) acting as effective transport boundaries (*7*). Whether this assumption holds in the presence of energetic submesoscale turbulence remains largely untested by direct observations. Growing observational and modeling evidence indicates that many mesoscale eddies deviate substantially from this idealized picture and instead undergo continuous erosion of coherence (*8*, *9*). Ageostrophic submesoscale processes at *O*(0.1 to 10) km can generate intense lateral stirring and mixing that promote tracer leakage between the eddy core and its periphery, as well as vertical exchange (*10*–*12*). Such leakage points to a critical mismatch between the classical geostrophic view of coherent mesoscale eddies and the reality of their leaky evolution, with profound consequences for nutrient supply, organic matter pathways, and pollutant fate in the ocean (*13*–*16*).

Despite their recognized importance and ubiquitous surface signatures evident in ocean color imagery, direct observation of submesoscale flows remains a major challenge because of their small spatial scales, transient nature, and short lifetimes (*17*). Several ambitious field programs have advanced understanding of submesoscale dynamics (*18*–*23*), yet resolving the velocity field and associated dispersion at submesoscales, particularly within mesoscale eddies, has remained elusive. Recent satellite missions such as the Surface Water and Ocean Topography (SWOT) mission provide unprecedented submesoscale-permitting measurements of sea surface height (SSH) (*24*–*26*), but their intermittent sampling and the difficulty of inferring ageostrophic velocities from SSH limit their ability to directly quantify submesoscale stirring and cross-eddy exchange. In contrast, clustered drifter deployments offer a powerful Lagrangian approach for directly capturing submesoscale motions through particle trajectories (*20*, *27*–*29*). However, most dispersion studies yield bulk statistical measures and cannot isolate the specific dynamical processes, such as submesoscale convergence (*16*) and radial stirring, responsible for eddy leakage. Addressing this gap requires purposefully designed field experiments that explicitly target submesoscale dynamics within mesoscale eddies.

Here, we present clustered drifter experiments conducted within an anticyclonic mesoscale eddy, providing high-resolution Lagrangian measurements in both the mixed layer and subsurface. This observational strategy enables direct resolution of submesoscale turbulence embedded within a mesoscale eddy—an essential but previously poorly observed process of eddy dynamics. Using an eddy-centered reference frame, we quantify submesoscale-driven dispersion and diffusivity and diagnose the associated ageostrophic flow characteristics. The observations demonstrate that submesoscale turbulence actively drives tracer exchange between the eddy core and its periphery, continuously eroding mesoscale eddy coherence. These results challenge the long-standing view of mesoscale eddies as materially conservative vortices in geostrophic balance and identify a key physical mechanism for eddy leakage, with broad implications for ocean transport, biogeochemical cycling, and pollutant fate.

## RESULTS

### Uniquely designed drifter-cluster experiments

As part of a field campaign targeting eddy dynamics in the South China Sea (SCS), we conducted uniquely designed clustered drifter deployments to directly observe submesoscale turbulence. A total of 242 drifters were strategically released around and within an energetic anticyclonic mesoscale eddy identified from daily satellite-based SSH fields (Fig. 1). This eddy exhibited a sea level anomaly of ∼30 cm, had persisted for more 7 weeks before our first deployment on March 23, and propagated southwestward at an average speed of ∼8 km/day. Throughout the experiments, the eddy maintained a relatively stable SSH structure, providing an ideal environment to examine submesoscale processes embedded within the seemingly coherent mesoscale structure (based on altimetry SSH contours).

**Fig. 1.**
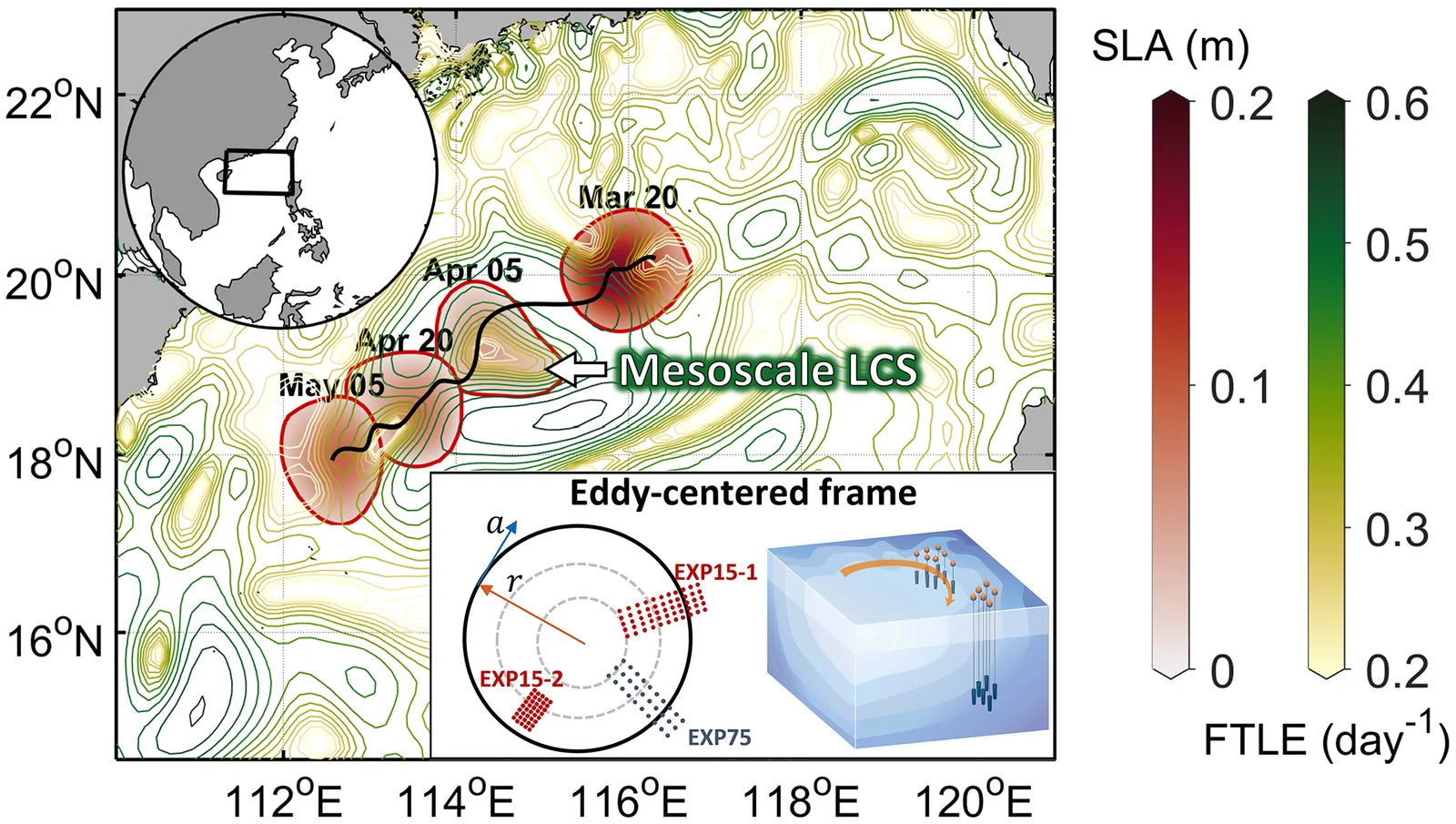
Map of the observed mesoscale eddy in the South China Sea and the configuration of the drifter-cluster experiments. The shaded red patches indicate the satellite-derived sea level anomaly (SLA) of the eddy at selected times, highlighting the apparent coherence of the eddy core, while the black curve traces the eddy center during the observational period. Colored contours show the forward FTLE field computed from daily geostrophic velocities on April 5, highlighting the repelling Lagrangian coherent structure (LCS) of the eddy. The lower-right inset schematically illustrates the deployment configurations of two 15-m-drouged drifter-cluster experiments (EXP15-1 and EXP15-2) and one 75-m-drouged experiment (EXP75) in the idealized eddy-centered reference frame. The radial (r) and azimuthal (a) directions are indicated by arrows in the eddy-centered reference frame.

To characterize the mesoscale background, we computed a forward finite-time Lyapunov exponent (FTLE) field on April 5 using SSH-based geostrophic velocities. The forward FTLE map delineates repelling LCSs (*7*, *30*), revealing a low-FTLE eddy core encircled by closed FTLE contours—a hallmark of strong mesoscale coherence (highlighted by the rectangle in Fig. 1). Consistent with previous work (*31*), elevated FTLE ridges trace regions of intense mesoscale stretching, marking dynamical boundaries that, in the geostrophic framework, are expected to inhibit exchange between the eddy interior and exterior. However, because the FTLE field here is derived from daily geostrophic flow, it reflects only the mesoscale deformation and cannot resolve the divergence/convergence associated with submesoscale turbulence. The clustered drifter experiments were therefore designed specifically to detect these unresolved ageostrophic motions and quantify their role in weakening mesoscale coherence and enabling eddy leakage.

Using the SSH field as a reference, three closely spaced drifter clusters were deployed to resolve submesoscale motions at different depths. The deployment configuration is schematically illustrated in the inset of Fig. 1. Two clusters (EXP15-1 and EXP15-2) comprised 53 and 65 satellite-tracked drifters, respectively, each equipped with 15-m drogues to follow mixed-layer currents. The drifters were deployed with nominal initial spacings of ∼2.5 km (EXP15-1) and ∼0.6 km (EXP15-2) between adjacent pairs, enabling multi-scale sampling of submesoscale turbulence near the eddy periphery. The latter extremely dense cluster resolves ∼1-km-scale dispersion characterized by strong convergence/divergence, driven by submesoscale turbulence. A third cluster, EXP75, comprised 33 drifters equipped with 75-m drogues and was released along transects extending from the eddy core to its periphery, with a nominal initial spacing of ∼4 km. This experiment was specifically designed to observe submesoscale turbulence below the mixed layer (shallower than 40 m; fig. S1A). Owing to deflection of the 75-m tether during drifting, the drogue was maintained at an effective depth of approximately 60 to 70 m. In these three experiments, about 80% of the drifters returned continuous long-lived, high-quality trajectories, which are used for the analyses presented here. To our knowledge, this is the largest drifter experiment to date targeting a specific eddy designed to resolve submesoscale dynamics, and the largest drifter deployment with drogues spanning and extending below the mixed layer. The dense drifter array provides high-resolution velocity observations for quantifying submesoscale processes. Potential influences from wind-driven Ekman flows, near-inertial motions, and internal tides were carefully assessed, and the corresponding analyses are presented in the Supplemental Materials.

The unique design of these experiments offers several key advantages over previous Lagrangian studies:

1. It enables simultaneous observations of submesoscale dispersion in both the mixed layer and the subsurface of a mesoscale eddy, allowing assessment of effects of submesoscale stirring at different depths.

2. It allows decomposition of dispersion into radial and azimuthal components in a polar coordinate framework centered on the evolving eddy, thereby capturing anisotropic dispersion patterns.

3. It permits direct estimation of submesoscale dynamical quantities including components of vorticity, divergence, and strain by resolving local horizontal velocity gradients between closely spaced drifters.

Collectively, the experiments offer a rare opportunity to directly observe submesoscale turbulence and quantify its associated dispersion within a mesoscale eddy environment. Complementary temperature fields from CMEMS reveal spiral-shaped temperature fronts and filaments (|∇T|) that intrude into the eddy core, providing important clues for interpreting drifter dispersion and the observed submesoscale convergence and divergence patterns (Fig. 2). In addition, rapid, high-resolution TRIAXUS towed-body surveys yielded cross-eddy temperature and salinity sections (fig. S1), which both delineate the eddy periphery for targeted drifter deployment and expose submesoscale fronts in the eddy region.

**Fig. 2.**
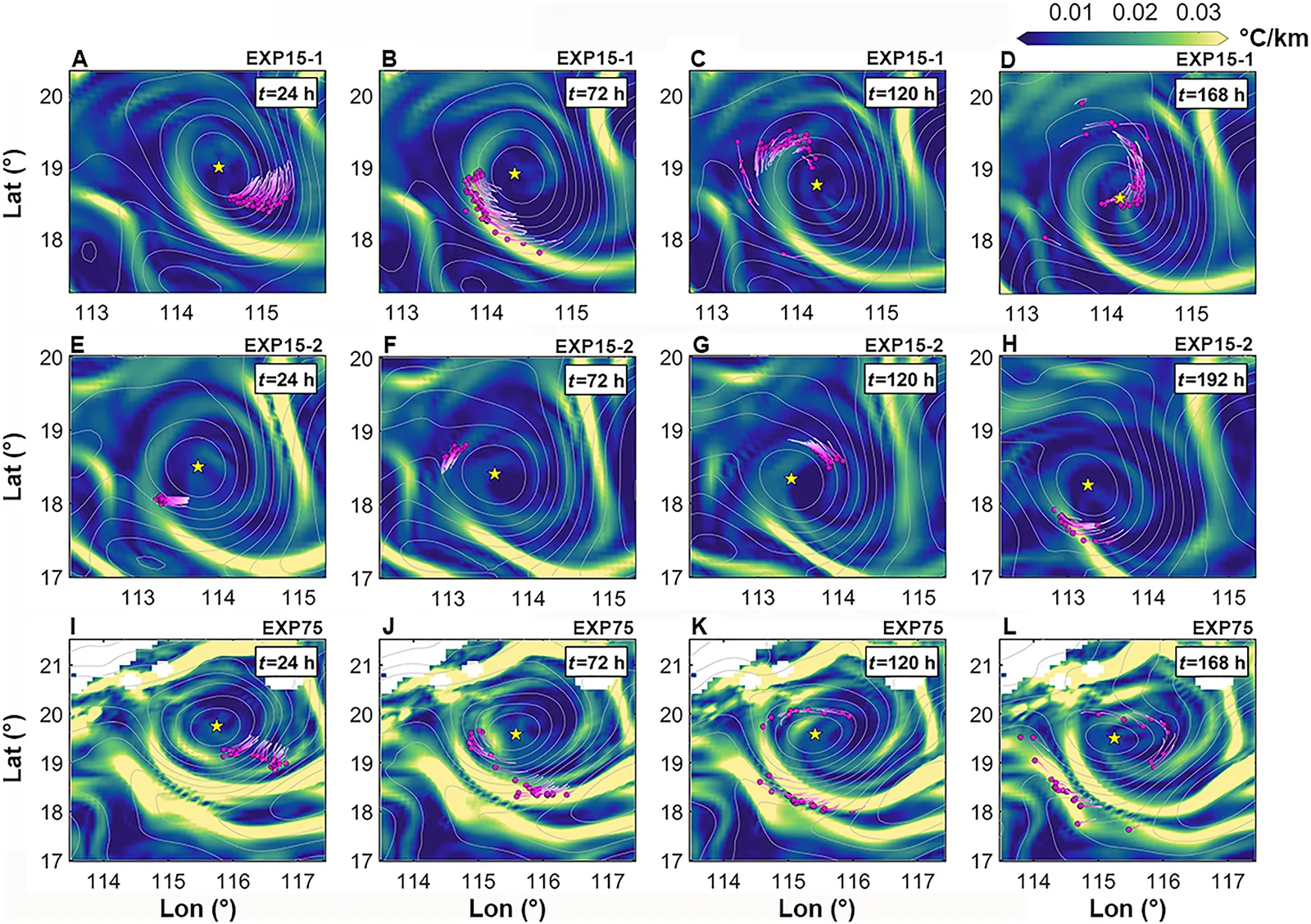
Drifter positions at selected times, with 18-hour trajectories shown as tails. The top row (**A** to **D**) depicts the tracer evolution in EXP15-1, the middle row (**E** to **H**) in EXP15-2, and the bottom row (**I** to **L**) in EXP75. Shading indicates temperature fronts calculated from CMEMS sea surface temperature (1/12° horizontal resolution), with sea surface height (SSH) contours overlaid. The star marks the eddy center in each plot. For each experiment, *t* = 0 hours corresponds to the reference time: 0700 April 6 for EXP15-1, 0300 April 16 for EXP15-2, and 1100 March 24 for EXP75.

### Clustering and dispersing features

Figure 2 presents the evolution of the three drifter clusters (colored magenta) during the first several days after deployment, when they remained closely spaced and largely confined within the mesoscale eddy. The time-lapse snapshots, with trajectory tails representing the preceding 18 hours of motion, visualize the combined influence of mesoscale rotation and embedded submesoscale turbulence on cluster evolution. The clustering and dispersal behaviors observed in each experiment reveal localized and transient drifting patterns that shed light on the underlying dynamics.

In EXP15-1 (upper row in Fig. 2), the near-surface drifters were deployed from the vicinity of the eddy core to its periphery. During the early phase, the drifter motions were highly controlled by the eddy flow, exhibiting anticyclonically rotational motion, with most drifters showing a slight inward migration (Fig. 2A). Around *t* = 72 hours, the drifters were abruptly captured by a sharp front that rapidly reorganized the drifters into a narrow filament aligned with the winding frontal axis (Fig. 2B). This clustering was accompanied by drifter trajectories crossing the mesoscale SSH contours, rather than remaining aligned with the geostrophic streamlines. This is confirmed by a more quantitative assessment of convergence in the subsequent analyses of relative dispersion. The drifters subsequently intruded toward the eddy core along the front, highlighting an active exchange pathway. As the cluster moved to the northwest of the eddy, radial spreading clearly intensified, while flow velocities weakened near the eddy center (shorter trajectory tails in Fig. 2C). By *t* = 168 hours, many drifters reached the eddy core region rather than returning to their initial positions after one azimuthal circuit—behavior inconsistent with purely mesoscale geostrophic advection (Fig. 2D). These observations provide direct observational evidence of tracer intrusion into the eddy core along the front. Only a few outer drifters dispersed away from the main cluster and were entrained by nearby submesoscale eddies—such as the northernmost drifter, which was spinning within a small-scale anticyclone (Fig. 2D). Examining the synthetic trajectories based on geostrophic flows reveals similar entraining features (fig. S2A), suggesting that during that period mesoscale geostrophic strain largely organized the entrainment and filament geometry (*9*). However, as illustrated by the relative dispersion analyses in the following section, the geostrophic mesoscale flow alone cannot reproduce the observed strong submesoscale convergence, clustering, and associated ageostrophic dispersion processes.

The drifters of EXP15-2 (middle row in Fig. 2), deployed to target submesoscale dynamics near the eddy periphery, exhibited dispersion characteristics markedly different from those in EXP15-1. Shortly after deployment, the drifters rapidly converged into a compact cluster (Fig. 2E). During the first few days, they remained densely packed while circulating around the eddy, with most nearby pairwise separations below 3 km. Dispersion became pronounced in the northwest sector, where the eddy’s coherent structure weakened, as indicated by the SSH contours (Fig. 2F). Unlike in EXP15-1, the drifters were not transported into the eddy core but instead spread gradually. This difference may be attributed to the relatively stable and symmetric state of the eddy during this period, when the frontal intrusion was less developed and the cluster was only partially influenced by the convergence zone. These observations provide an ideal case for quantifying submesoscale dispersion and estimating lateral diffusivity near the eddy periphery, as examined in the following sections.

In the subsurface, the drifters were initially deployed to the southeast of the eddy (Fig. 2I). Soon after, this relatively sparse cluster split into two groups, separated roughly by an SSH contour that acted as a dynamical boundary (Fig. 2J). One group remained within the closed contour and continued to circulate around the eddy. Despite their limited number of drifters, this group exhibited pronounced azimuthal and radial dispersion, revealing active submesoscale turbulence in the subsurface layer. The other group, initially positioned near the outer ring of the eddy, experienced pronounced outward transport (Fig. 2K). During this phase, these drifters’ motions were likely strongly modulated by frontal submesoscale instabilities during the interaction between the mesoscale anticyclone and an adjacent cyclone to the east, as suggested by the tightly packed SSH contours between the two eddies. These processes produced notable radial dispersion. As the drifters in this group migrated southward along the anticyclone’s flank, most eventually escaped the eddy’s influence and dispersed away (Fig. 2L), indicating a pathway of eddy leakage facilitated by submesoscale processes during the cyclone-anticyclone interaction. A few drifter pairs, however, maintained close separations, likely trapped within localized submesoscale turbulence just outside the eddy periphery.

### Relative dispersion

To quantify the evolution of drifter dispersion, relative separation *D*_i_(*t*) is computed for each drifter pair, and the relative dispersion *D*^2^(*t*) is defined as the mean of the squared separations over all selected pairs (see Materials and Methods). This metric characterizes the rate at which tracers spread apart and provides the basis for estimating relative diffusivity. Note that, within the eddying flow, drifters were experiencing shear dispersion likely resulting in pronounced anisotropy between azimuthal and radial spreading. Accordingly, *D*_i_(*t*) is decomposed into azimuthal (*D*_i,a_) and radial (*D*_i,r_) components with respect to the time-varying eddy center (inset of Fig. 1). Although the decomposition relies on a circular approximation of the eddy and the relative dispersion for each component is defined as ensemble means after projection, our analyses indicate that it provides robust and physically meaningful characterization of anisotropic dispersion (see Materials and Methods). Furthermore, a comparison with an alternative decomposition based on directions across and along SSH contours yields qualitatively consistent results (fig. S3), supporting the robustness of the radial-azimuthal framework. Here, we place particular emphasis on the radial component, which directly governs cross-eddy exchange and is therefore purely associated with submesoscale processes. This is also supported by the velocity correlation analysis (*29*, *32*): A low correlation in the radial direction is observed, indicating rapid separation by submesoscale turbulence, whereas the azimuthal velocity component—strongly influenced by the mesoscale eddy circulation—exhibits high velocity correlation (fig. S4). In addition, relative dispersion derived from synthetic trajectories advected by geostrophic velocities is also shown for comparison (Fig. 3). Although the magnitude of the coarse geostrophic velocities is weaker than that experienced by the drifters (*33*), these synthetic trajectories faithfully capture the evolving mesoscale flow structure (fig. S2A), particularly during the early time of EXP15-1 when the eddy is less stable. The contrast between the synthetic and observed dispersions can highlight the critical role of submesoscale turbulence in enhancing relative dispersion beyond that attributable to mesoscale geostrophic motions alone.

**Fig. 3.**
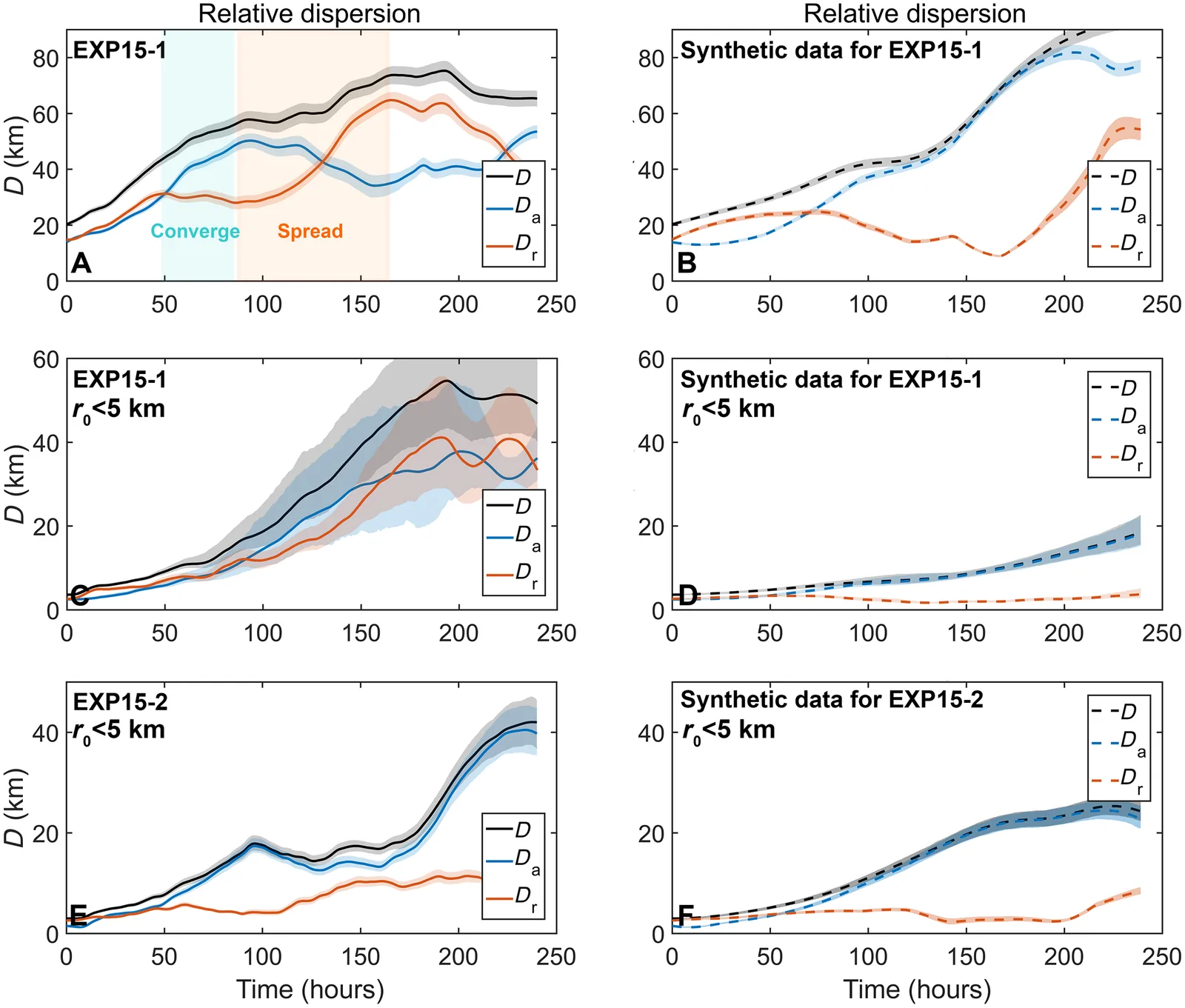
Time evolution of relative dispersion D(*t*) and its decomposed azimuthal (*D*_a_) and radial (*D*_r_) components. (**A**) Relative dispersion derived from all drifter pairs in EXP15-1. (**B**) Relative dispersion derived from synthetic trajectories based on geostrophic flows for EXP15-2. In (A), light peacock blue and orange shading indicate periods of net convergence and spreading the drifter cluster, respectively. *D*, *D*_a_, and *D*_r_ computed using only drifter pairs with initial separations *r*_0_ < 5 km are shown for (**C**) EXP15-1, (**D**) synthetic data for EXP15-1, (**E**) EXP15-2, and (**F**) synthetic data for EXP15-2. The shaded envelopes denote 95% confidence intervals estimated via bootstrapping.

As shown in Fig. 3, the dispersion increases rapidly with time and exhibits clear anisotropy between the azimuthal and radial directions. In EXP15-1, *D*_r_ temporarily decreases between 48 and 72 hours, coinciding with the frontal convergence event (highlighted by the light peacock blue shading in Fig. 3A). This is followed by a period of rapid radial spreading (highlighted by light red shading in Fig. 3A). The enhanced radial dispersion during this period coincides with a suppression of azimuthal dispersion, manifested as increasing *D*_r_ and decreasing *D*_a_. As mentioned in the last section, this is because the dispersion during this period is highly controlled by the frontal intrusion toward the eddy core (recall Fig. 2, C and D). A similar feature is evident in the synthetic trajectories for EXP15-1 (fig. S2A) and the associated relative dispersion (Fig. 3B), although with a delay of about 50 hours. In the synthetic case (Fig. 3B), the relative dispersion is dominated by the azimuthal component, primarily reflecting shear dispersion driven by the mesoscale geostrophic flows. However, small differences between the observed and synthetic velocity fields are progressively amplified over time, leading to substantially different tracer pathways and transport outcomes. Additional synthetic experiments initialized from the observed drifter locations at different times illustrate the sensitivity of tracer fate to submesoscale motions (fig. S2, C and D).

To focus on submesoscale dispersion, we further analyzed the evolution of relative dispersion for subsets of drifter pairs with initial separations *r*_0_ < 5 km in each experiment (Fig. 3, C to F). Although fewer pairs are available (85 and 243 pairs for EXP15-1 and EXP15-2, respectively), the subsets more accurately capture the influence of submesoscale dynamics, particularly during the early tens of hours. As expected, the resulting dispersion magnitudes are much smaller and are primarily attributable to submesoscale motions. In EXP15-1, comparison between Fig. 3C (drifter pairs with *r*_0_ < 5 km) and Fig. 3A (all drifter pairs) reveals markedly different behavior for submesoscale dispersion: Both the azimuthal (*D*_a_) and radial (*D*_r_) components remain small (*D* < 10 km) before *t* = 90 hours and appear more isotropic. Synthetic trajectories based solely on geostrophic flows substantially underestimate the observed dispersion (Fig. 3D versus Fig. 3C). Particularly, the radial component (*D*_r_) is strongly enhanced by submesoscale motions, and even the azimuthal component (*D*_a_) shows clear amplification. For pure geostrophic flow, the submesoscale dispersion is remarkably weaker than that by mesoscale flow, which can also disperse tracers (Fig. 3, B and D). These results highlight the essential role of submesoscale turbulence in dispersing tracers near the mesoscale eddy periphery.

For the dense deployment in the eddy periphery (EXP15-2), the radial component *D*_r_ remains relatively weak (below 10 km) throughout the first 10 days, while the azimuthal component *D*_a_ begins to grows rapidly after about 50 hours (Fig. 3E). This behavior is owing to the initially tight spacing of these drifters. Unlike EXP15-1, relative dispersion in EXP15-2 is mostly dominated by the azimuthal component, indicating weaker radial dispersion for EXP15-2, as a result of more stable eddy state during this experiment. Comparison with that of synthetic trajectories shows only limited enhancement in both radial and azimuthal dispersion, suggesting that submesoscale motions during EXP15-2 make a relatively modest contribution to tracer spreading within the eddy. Because the drifters in EXP75 separated into two distinct groups, relative dispersion in this experiment does not provide a fair or representative measure of the dispersion characteristics. Nevertheless, submesoscale turbulence continued to influence drifter spreading, as evidenced by the dispersive features (recall Fig. 2, I to L).

### Enhanced flow deformation by submesoscale processes

To further quantify the deformation and stirring induced by submesoscale motions, we computed FTLE_drifters_ from hourly drifter trajectories using pairs with initial separations *r*_0_ < 5 km. Because the drifters typically move less than 2 km within 1 hour, the resulting changes in pair separation remain within the submesoscale range. By definition (see Materials and Methods), FTLE_drifters_ quantifies the finite-time stretching or compression of nearby drifter pairs over a 1-hour interval. It therefore characterizes the intensity of evolving submesoscale deformation (Fig. 4) and serves as a measure of stirring associated with submesoscale turbulence. In contrast to FTLE_geo_, which is computed from daily SSH-derived geostrophic velocities and primarily reflects deformation by the mesoscale flow field (the contours in Fig. 1), FTLE_drifters_ captures the combined effects of mesoscale and unresolved ageostrophic motions, including submesoscale frontal processes. As shown in Fig. 4, FTLE_drifters_ values are almost two to three times larger than FTLE_geo_, indicating substantially enhanced stretching and compression at submesoscales beyond that expected from the mesoscale geostrophic circulation alone. These results highlight the important role of submesoscale ageostrophic motions in intensifying local deformation and disrupting mesoscale eddy coherence. A representative example occurs around *t* = 50 hours in EXP15-1. The previously described frontal event produces strong radial contraction of drifter pairs, resulting in negative FTLE_drifters_ values (highlighted by the black dashed rectangles in Fig. 4, A and D), while simultaneous along-front stretching generates adjacent positive FTLE_drifters_ peaks. Together, these signals imply intense filamentation associated with the frontal dynamics. Such localized stretching and compression are largely absent from FTLE_geo_, which is derived from the smoother geostrophic velocity field. Although stretching and compression occur intermittently, the mean FTLE_drifters_ remains predominantly positive (Fig. 4D), indicating that submesoscale motions generally enhance particle separation and stirring. The elevated FTLE_drifters_ near the eddy boundary, compared with FTLE_geo_ (Fig. 1), suggests that submesoscale motions substantially increase the deformation of material boundaries surrounding the eddy core, thereby facilitating cross-eddy exchange and tracer leakage. Note that the averaged FTLE_drifters_ exhibits a decreasing trend over time, likely reflecting a weakening eddy during the experiments, as indicated by the SLA field in Fig. 1.

**Fig. 4.**
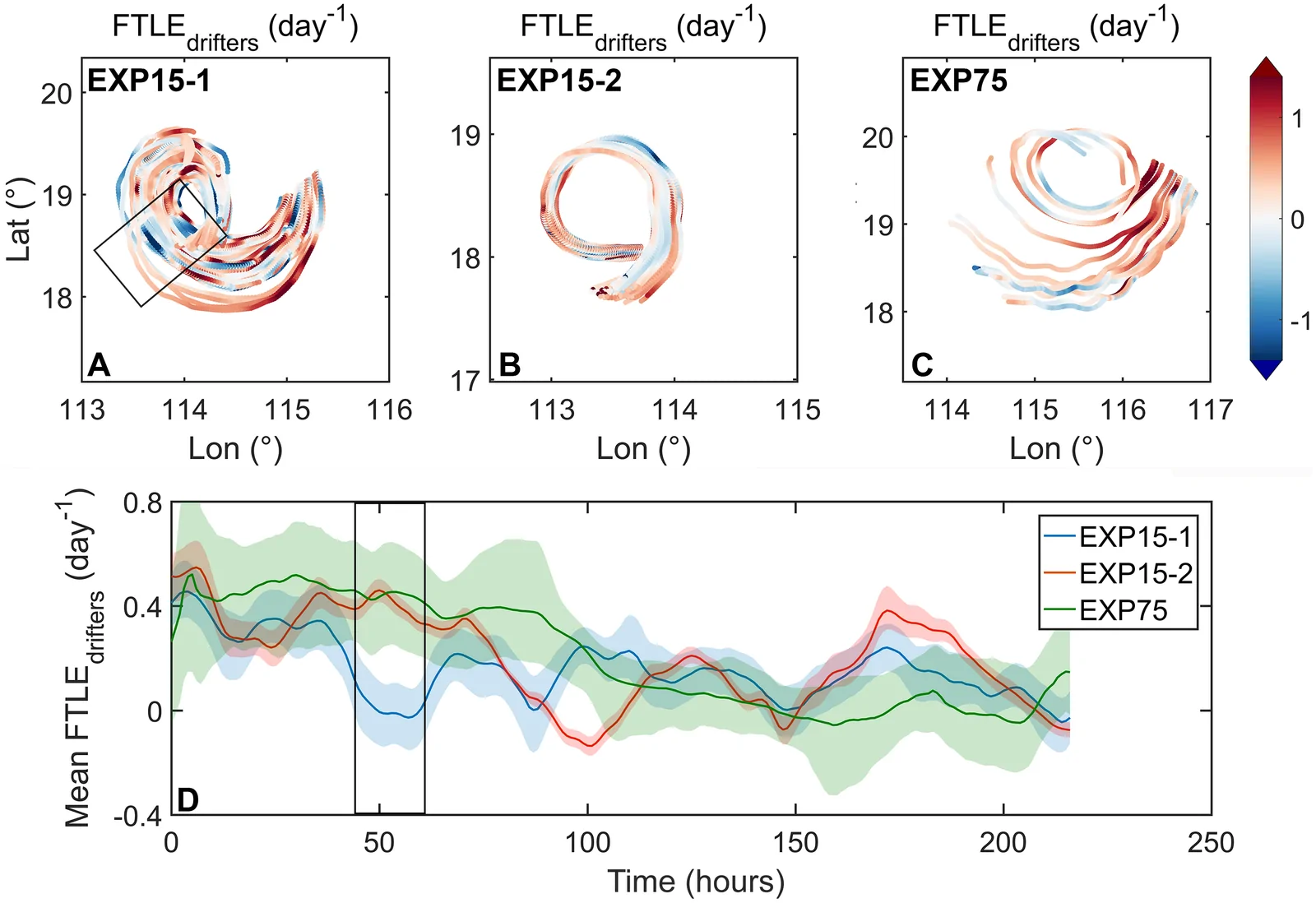
FTLE computed from hourly trajectories of nearby drifter pairs (FTLE_drifters_). FTLE_drifters_ are shown for (**A**) EXP15-1, (**B**) EXP15-2, and (**C**) EXP75 in the early 220 hours after deployment. (**D**) Temporal evolution of the mean FTLE_drifters_ for each experiment. The shaded envelopes denote 95% confidence intervals estimated via bootstrapping.

### Submesoscale diffusivity

In this section, relative diffusivity (*K*), defined as the rate of change of relative dispersion (see Materials and Methods), is used to quantify submesoscale dispersion in the three experiments. To isolate the submesoscale diffusivity (*K*_sm_), a prescribed submesoscale threshold of 10 km is applied when selecting the drifter pairs for analysis. With this criterion, the estimated diffusivity is primarily associated with submesoscale dynamics rather than mesoscale stirring. Furthermore, decomposing *K*_sm_ into radial (*K*_sm,r_) and azimuthal (*K*_sm,a_) components enables a focused examination of the radial component, which reflects inward or outward tracer spreading relative to the eddy center and directly controls cross-eddy exchange. Compared with bulk diffusivity estimates like the mixing length framework (*34*, *35*), this pair-based approach provides a more direct and precise quantification of submesoscale diffusivity.

The relative diffusivity as a function of pair separation (*r*) for the three experiments (Fig. 5A) shows that, across the submesoscale range, diffusivity increases approximately as *r*^2^, consistent with a nonlocal regime. This indicates that pair dispersion at kilometer scales is primarily controlled by mesoscale strain rather than by locally generated submesoscale turbulence (*29*). Nevertheless, the observed diffusivity is substantially enhanced relative to that derived from purely geostrophic flows (Fig. 5B), indicating that submesoscale processes act as intermittent amplifiers of deformation, particularly in frontal regions. The decomposition into radial and azimuthal components further reveals a weak anisotropy within this regime: Azimuthal diffusivity is slightly larger than radial diffusivity (Fig. 5C). This implies that along-eddy shear and rotational stirring dominate particle spreading, while cross-eddy transport is comparatively more constrained. Notably, the drifters in EXP75 also experienced energetic diffusivity near 10 km (purple line in Fig. 5A), suggesting the presence of submesoscale dispersion in the subsurface layer of the eddy. As discussed above, interactions between the anticyclonic eddy and the adjacent cyclonic eddy to the east may contribute to the generation of these submesoscale motions.

**Fig. 5.**
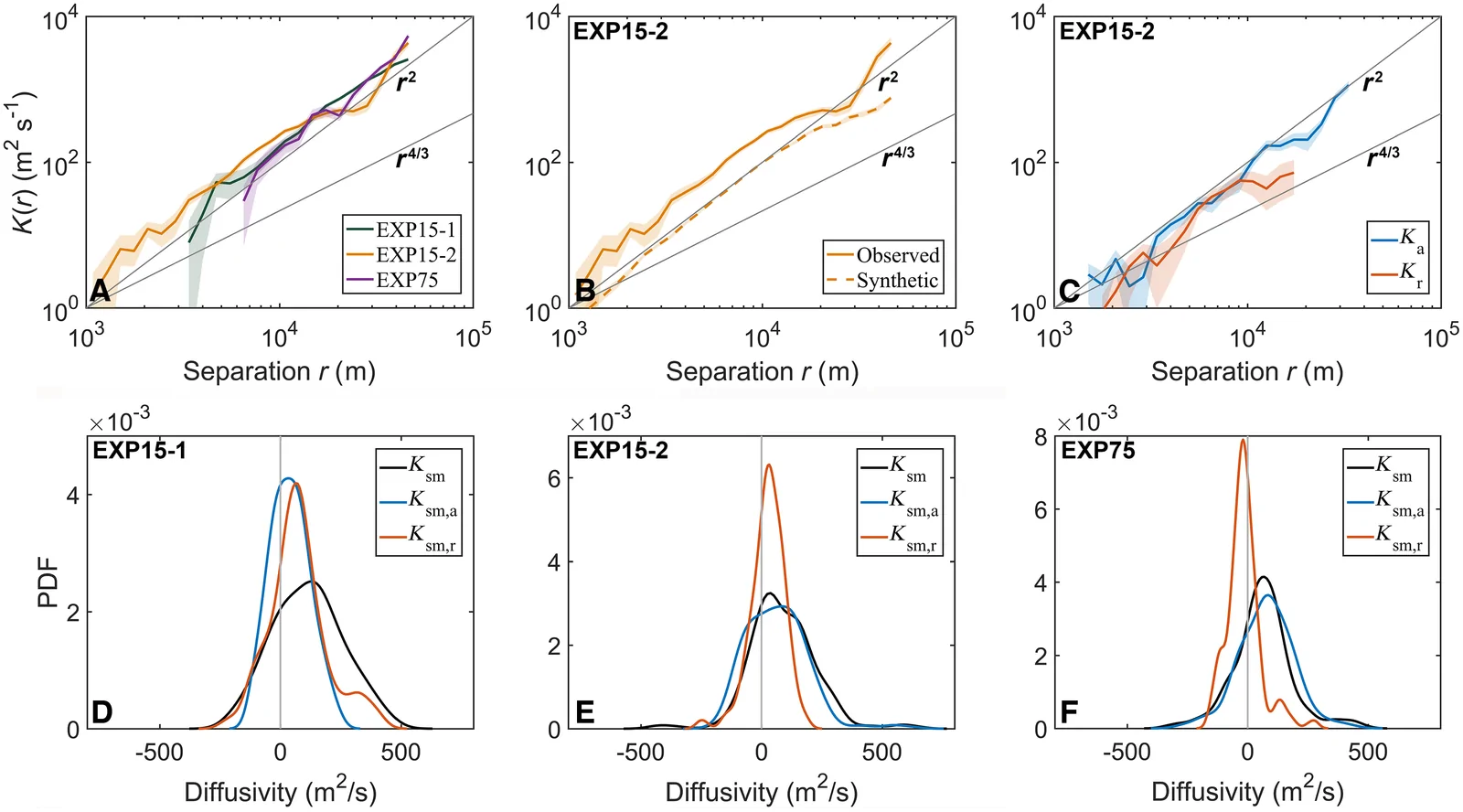
Diffusivity as a function of separation and statistics of diffusivity decomposition into azimuthal and radial components. (**A**) Scale-dependent relative diffusivity *K* as a function of separation *r*. (**B**) Comparison of observed and synthetic data–based *K* for EXP15-2. (**C**) Comparison of azimuthal (*K*_a_) and radial (*K*_r_) components for *K*. The gray lines indicate *r*^2^ (in a nonlocal regime) and *r*^4/3^ (in a local regime). The shaded envelopes denote 95% confidence intervals estimated via bootstrapping. PDF of relative diffusivity estimated using drifter pairs with separations below 10 km for (**D**) EXP15-1, (**E**) EXP15-2, and (**F**) EXP75.

Figure 5 also shows the probability distribution functions (PDFs) of submesoscale relative diffusivity over early 10 days after deployment for each experiment (bottom raw). Notably, negative diffusivity values occur frequently, indicating intermittent convergence associated with submesoscale processes. In EXP15-1, submesoscale diffusivity predominantly ranges between −300 and 600 m^2^/s (Fig. 5D). The PDFs of *K*_sm_, *K*_sm,r_, and *K*_sm,a_ all show clear positive skewness, indicating enhanced spreading driven by submesoscale turbulence. The mean absolute diffusivities are 148.1, 109.0, and 71.9 m^2^/s for the total (|*K*_sm_|), radial (|*K*_sm,r_|), and azimuthal (|*K*_sm,a_|) components, respectively. During this period, the radial component is comparable to, and even exceeds, the azimuthal component in magnitude, highlighting the importance of submesoscale processes in facilitating cross-eddy transport. In EXP15-2 where drifters were deployed with smaller initial separations, the resulting diffusivity values are generally reduced, mostly ranging between −200 and 300 m^2^/s (Fig. 5E), consistent with previous observational studies (*29*). The mean absolute diffusivity is estimated as 121.4, 58.7, and 106.4 m^2^/s for *K*_sm_, *K*_sm,r_, and *K*_sm,a_, respectively. The lower radial diffusivity compared with EXP15-1 likely results from the smaller pair separations, which preferentially sample weaker submesoscale dispersion. In addition, the mesoscale eddy during EXP15-2 was more stable and symmetric as indicated by the nearly circular SSH contours (middle row in Fig. 2), conditions that are generally less favorable for strong submesoscale activity. In EXP75, submesoscale dispersion is expected to be largely weakened in the subsurface layer, particularly in the radial direction. Nevertheless, submesoscale diffusivity remains appreciable, with a magnitude of 105.2 m^2^/s, although dispersion is dominated by the azimuthal component (Fig. 5F). It should be noted that during this experiment, a subset of drifters drifted away from the eddy after several days, making the eddy-centered decomposition less appropriate and potentially affecting the interpretation of the radial and azimuthal components.

### Potential submesoscale dynamics

Using the observed velocities, the clustered drifter arrays allow for direct calculation of key kinematic quantities, including vorticity (ζ), divergence (δ), and strain rate (*S*), which characterize submesoscale turbulence (*36*, *37*). These quantities are computed using a four-point group method (see Materials and Methods). To specifically target submesoscale motions, we retain only drifter groups that remain within 5 km in both the zonal (*x*) and meridional (*y*) directions. Figure 6 presents the statistics of ζ, δ, and *S* normalized by the local Coriolis parameter *f*, and shows divergence conditioned on the vorticity-strain space (*38*). This facilitates interpretation of the submesoscale regimes sampled during the three experiments.

**Fig. 6.**
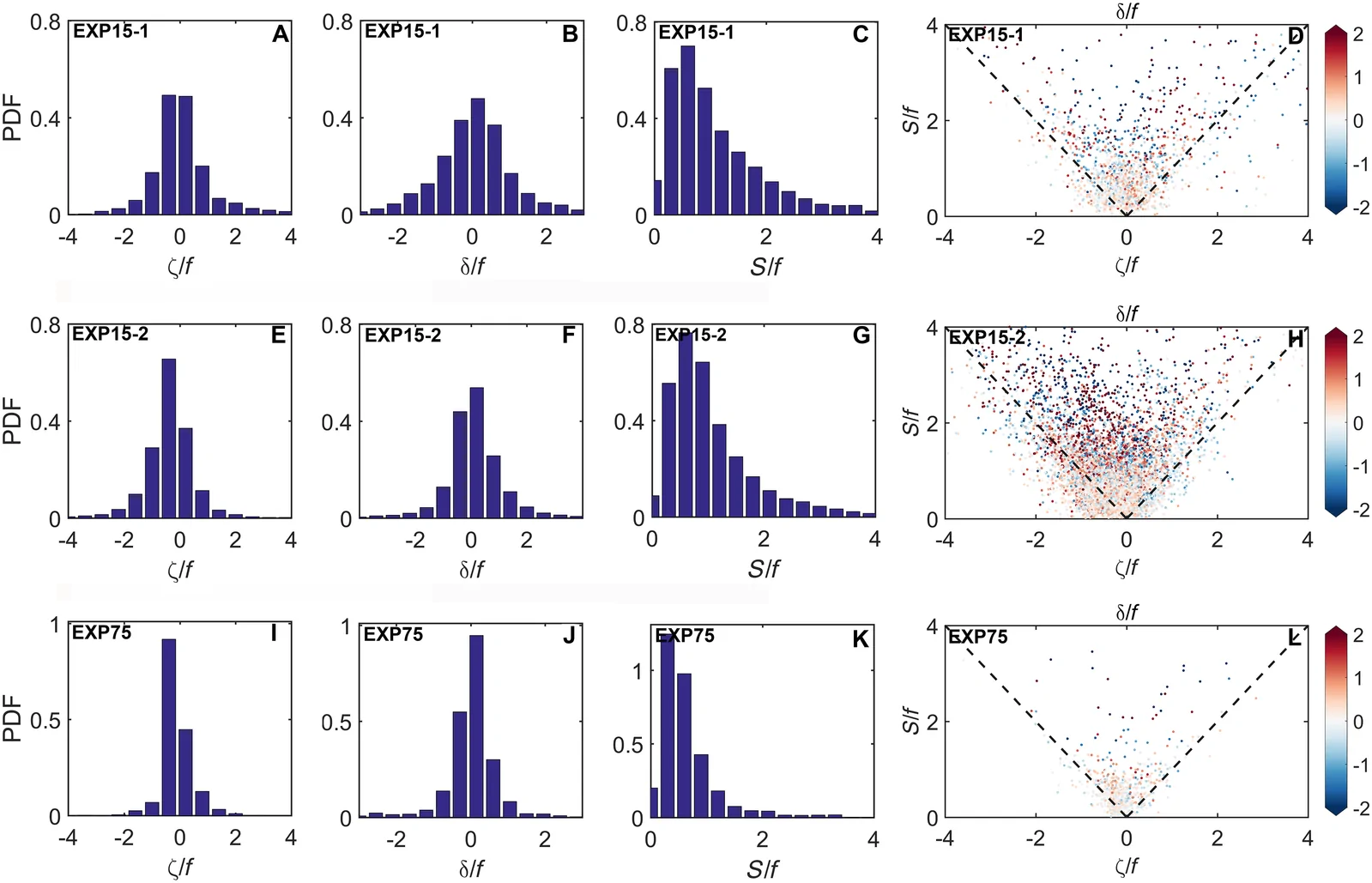
Normalized vorticity (ζ), divergence (δ), and strain rate (*S*) derived from submesoscale four-point drifter groups, illustrating submesoscale dynamics. (**A** to **C**) Statistics of ζ/*f*, δ/*f*, and *S*/*f*, and (**D**) divergence conditioned on the vorticity-strain space from EXP15-1. (**E** to **G**) Statistics of ζ/*f*, δ/*f*, and *S*/*f*, and (**H**) divergence conditioned on the vorticity-strain space from EXP15-2. (**I** to **K**) Statistics of ζ/*f*, δ/*f*, and *S*/*f*, and (**L**) divergence conditioned on the vorticity-strain space from EXP75.

For all deployments, the drifters captured both strong cyclonic and anticyclonic vorticity. In the mixed layer, the relative vorticities (|ζ/*f* |), a proxy of Rossby number, can reach values as large as 4 (Fig. 6, A and E). These pronounced ageostrophic motions are accompanied by strong divergence and convergence (Fig. 6, B and F), resulting in the enhanced dispersion signatures described earlier. Notably, large negative vorticity values occur nearly as frequently as positive values—an unusual feature in the ocean, given that ζ/*f* < −1 is often indicative of centrifugal instability under idealized assumptions. Recalculating the vorticity using low-frequency filtered velocities yields strong negative ζ/*f* < −1 (fig. S8), indicating that this signal is robust and not primarily caused by wave motions. Here, the prevalence of intense negative vorticity is more likely attributed to strain-dominated frontal dynamics and submesoscale filamentation, rather than to submesoscale vortices, which are not evident in the drifter trajectories (Fig. 2). In addition, Lagrangian sampling can preferentially concentrate drifters within convergent frontal regions, potentially producing vorticity statistics that differ substantially from Eulerian observations (*39*), particularly in strongly convergent frontal environments. This interpretation is further supported by the joint vorticity-strain distributions (rightmost column in Fig. 6), which indicate that the sampled submesoscale flows predominantly occupy a strain-dominated regime (*S* > |ζ| for |ζ/*f* | > 1), rather than vorticity-dominated (*S* < |ζ|) or shear-dominated (*S* ≈ |ζ|) regimes. Such a strain-dominated state is characteristic of active frontogenesis, in which horizontal strain sharpens lateral density gradients and promotes the formation of submesoscale fronts. This is consistent with the sharp density fronts observed by the towed-body measurements (fig. S1). These results suggested that negative vorticity may be substantially underestimated in model-based studies, for example, in Balwada *et al*. (*38*), as submesoscale fronts are typically much narrower than the kilometer-scale resolution of most ocean models (*40*).

Although the subsurface flow is dynamically more balanced overall (low vorticity), the drifters in EXP75 nonetheless recorded pronounced ageostrophic motions with |ζ/*f* | > 1 (Fig. 6I) and strong strain rates (Fig. 6K), implying the presence of active submesoscale turbulence below the mixed layer. This likely arises from the relatively weak stratification within the eddy interior (fig. S1C), produced by the downward displacement of isopycnals in the anticyclonic eddy. Reduced stratification lowers the local Richardson number, creating conditions favorable for submesoscale instabilities. When combined with sufficiently strong horizontal strain, frontogenesis can be sustained even at depth. In addition, it is important to note that internal wave motions in the subsurface may alias the derived flow gradients and partially influence the distributions in vorticity-strain space (*41*, *42*). These results provide compelling evidence that submesoscale turbulence within mesoscale eddies both in the mixed layer and below it arises from multiple interacting mechanisms (*19*) and is capable of driving vertical tracer exchange (*12*, *43*).

## DISCUSSION

Direct observations of oceanic submesoscale flows remain exceedingly rare because of their small spatial scales, transient nature, and short lifetimes (*17*). Through purposefully designed, high-resolution drifter-cluster experiments, this study provides direct Lagrangian observations of submesoscale turbulence embedded within an anticyclonic mesoscale eddy. The relatively steady rotational background flow of the eddy allowed closely spaced drifters to remain in close proximity, thereby resolving transient ageostrophic motions at submesoscales. These experiments captured both clustering and dispersal of drifters in the mixed layer and subsurface, revealing clear signatures of submesoscale divergence and convergence. The simultaneous measurements enabled a rare, fully in situ characterization of submesoscale dynamics within a mesoscale eddy. The observations reveal energetic ageostrophic motions with large Rossby numbers (|ζ/*f*|), which drive vigorous stirring and dispersion at submesoscales, facilitating lateral tracer exchange between the eddy core and its periphery. Together, these results demonstrate that submesoscale turbulence actively disrupts mesoscale coherence, directly challenging the conventional geostrophic view of mesoscale eddies as dynamically isolated, conservative vortices. Consequently, transport frameworks based on persistent LCS may fail under such conditions, as submesoscale turbulence introduces irreversible leakage and limits the predictability of tracer fate across oceanic and environmental applications. It should be noted that these results do not invalidate the LCS theory, but demonstrate that its applicability is fundamentally limited in the presence of energetic submesoscale ageostrophic motions.

A central result of this study is the direct, quantitative estimate of submesoscale relative dispersion, which is often parameterized statistically by a relative diffusivity. The inferred submesoscale diffusivity ranges between *O*(10 to 100 m^2^ s^−1^), consistent with previous estimates but here obtained from direct, process-resolving observations. Lower diffusivity emerges at smaller submesoscales (EXP15-2 compared with EXP15-1), consistent with enhanced intermittency and weaker dispersion at finer scales (*44*). Over short time windows (hours), submesoscale motions generate both divergence and convergence, yielding comparable diffusivity in both the radial and azimuthal directions (Fig. 5). However, within the eddy environment, submesoscale dispersion is strongly coupled to background mesoscale rotation; azimuthal dispersion is readily amplified by mesoscale stretching and shear dispersion (as shown in Fig. 3). Notably, although mesoscale geostrophic flows can also contribute to radial dispersion through mesoscale deformation, they do not effectively contribute to cross-front tracer exchange—a process absent in purely balanced geostrophic dynamics. Enhanced radial dispersion around the eddy’s periphery enables irreversible leakage of water masses, heat, and biogeochemical tracers from the eddy core. This pathway provides a direct physical mechanism for eddy leakage.

Analysis of the submesoscale flow field indicates that these motions are highly ageostrophic, with intense horizontal divergence and convergence. Such divergence/convergence is dynamically significant because they are directly linked to vertical motions and exchange (*35*, *38*). The joint distributions of vorticity and strain indicate that the submesoscale flows within the eddy are predominantly in strain-dominated regimes, where frontogenesis and filamentation are active. These strain-driven processes sharpen density gradients, enhance lateral shear, and ultimately foster secondary circulations (*45*–*47*). Vertical motions have important implications for nutrient supply, ecosystem functioning, and carbon cycling. Additionally, this regime also implies a forward energy flux from the mesoscale to the submesoscale (*48*), serving as a plausible pathway for eddy depletion.

The intensity and structure of submesoscale motions are likely modulated by time-varying surface forcing, including wind stress and buoyancy fluxes. Variations in these forcings would alter mixed-layer depth, drive ageostrophic motion, and alter the strength and distribution of submesoscale turbulence and its dispersive effect. Additionally, the life stage of mesoscale eddies, whether growing, stable, and decaying, also exerts strong control over the intensity of submesoscale turbulence and the coherence of the mesoscale flow (*49*). During the mature phase, eddies usually exhibit greater symmetry and weaker mesoscale deformation, whereas enhanced strain during the growth and decay stages likely promote stronger submesoscale activity and increased tracer leakage.

Despite the advantages of the high-resolution drifter-cluster design, several limitations remain. The finite number of drifters constrains spatial sampling within the eddy. The drifters are not perfectly Lagrangian, so flow around and through the drogues surely affects the results somewhat. Although our analyses indicate that wave motions played a limited role in the submesoscale divergence estimated from our observations (figs. S7 and S8), potential aliasing by high-frequency internal waves, especially in the subsurface, cannot be fully excluded (*42*). While near-inertial motions are not strongly evident within the eddy (fig. S5), internal wave activities may nevertheless enhance shear, promote submesoscale instabilities, and indirectly contribute to lateral dispersion, especially below the mixed layer (*44*).

Overall, these observations provide new insight into the structure and dynamics of submesoscale turbulence within mesoscale eddies. The role of such turbulence in driving eddy leakage is a fundamental physical process that has not been widely appreciated. Although the present findings are based on a single observational case, the underlying mechanism is expected to operate broadly under favorable dynamical conditions, as analogous surface signatures associated with submesoscale activity are frequently observed in ocean color imagery (*50*). However, quantifying the global impact of submesoscale processes on eddy leakage remains a significant challenge. Accounting for these processes is essential for improving the representation of ocean stirring and material exchange in earth system and climate models.

## MATERIALS AND METHODS

### Observations

A field campaign was conducted from March 21 to Aril 18 to investigate submesoscale activity and its impacts within an energetic anticyclonic mesoscale eddy in the SCS. Guided by near-real-time SSH fields, we carried out an extensive suite of coordinated observations, including CTD stations, turbulence microstructure measurements, shipboard ADCP velocity profiles and air-sea fluxes, towed-body transects, glider surveys, and high-resolution drifter-cluster deployments. Together, these observations captured a comprehensive view of the physical and biogeochemical environment inside the eddy, resolving mesoscale structures, submesoscale fronts, vertical stratification, turbulent mixing, and associated ecological variability.

The drifters consisted of a 40-cm surface float attached to a cylindrical drogue. The design is compatible with standard GDP drifters (*51*) and provides a high drogue–to–exposed area ratio to minimize direct windage. The measured velocities therefore primarily represent Lagrangian currents at the drogue depth. Three drifter-cluster experiments were conducted to observe submesoscale turbulence in an anticyclonic mesoscale eddy. All drifters were deployed during the period of 20 March to 15 April 2025. Each drifter was equipped with a Global Positioning System (GPS) receiver with ∼10 m positional accuracy, transmitting locations every 10 min. To obtain dense, contemporaneous Lagrangian measurements at submesoscale separations, drifters were released in compact rectangular arrays (Fig. 1). This configuration allowed synoptic sampling of submesoscale motions while enabling rapid deployment from a single vessel and ensuring coverage from the eddy core to its periphery. Two types of drifters were used: 15-m-drouged drifters to sample mixed-layer flow and 75-m-drouged drifters to sample subsurface motions. After quality control, hourly positions and velocities were used for analysis of submesoscale dynamics.

During the cruise, we also collected high-resolution hydrographic observations using a TRIAXUS towed body, which measured conductivity, temperature, pressure, oxygen, and chlorophyll in the upper 250 m across the eddy. These data delineate the mesoscale structure and reveal embedded submesoscale fronts, aiding both the identification of the eddy periphery for targeted drifter deployment and the characterization of frontal features within and below the mixed layer (fig. S1).

To supplement the in situ measurements, we used daily temperature fields from the CMEMS 1/12° Operational Mercator Global Ocean Analysis and Forecast System (GOAFS) to illustrate the eddy structure and associated fronts. The surface temperature fields are calibrated using the Operational Sea Surface Temperature and Ice Analysis (OSTIA) product, which incorporates GHRSST satellite retrievals and in situ observations (fig. S6).

### FTLE

FTLE is a quantitative measure of the finite-time separation rate between initially adjacent fluid particles. For two particles with an initial separation vector $D\left(t_{0}\right)$, their separation after a finite time interval Δt becomes $D\left(t_{0}+∆t\right)$. The FTLE is defined as

$$\lambda \left(t_{0},\Delta t\right)=\frac{1}{|\Delta t|}\text{In}\left[\frac{|D\left(t_{0}+∆t\right)|}{|D\left(t_{0}\right)|}\right]$$ (1)

which represents the exponential growth (or decay) rate of particle separation over Δt. Positive FTLE values indicate local flow divergence or stretching, whereas negative values reflect convergence or compression. Here, the FTLE fields computed from daily geostrophic velocities (contours in Fig. 1) are used to characterize mesoscale LCSs. Differently, the FTLE_drifters_ derived from hourly drifter trajectories capture the instantaneous submesoscale motions, including frontal convergence/divergence and filamentation.

### Dispersion and diffusivity metrics

For each drifter pair *i* with positions **x**_*i*,1_(*t*) and **x**_*i*,2_(*t*) (subscripts 1 and 2 indicate the two members of the pair), the separation vector is

$$D_{i}\left(t\right)=x_{i,1}\left(t\right)-x_{i,2}\left(t\right)$$ (2)

Then, the relative dispersion is defined as the ensemble-mean squared separation

$$D{\left(t\right)}^{2}=\langle |D_{i}\left(t\right)\cdot D_{i}\left(t\right)|\rangle$$ (3)

where ⟨·⟩ denotes an average over selected drifter pairs in the cluster. The relative dispersion quantifies the growth of particle separations over time. To characterize anisotropy within the eddy, the relative dispersion is decomposed into radial and azimuthal components defined relative to the time-dependent eddy center. As illustrated in the inlet of Fig. 1, the two components are orthogonal. Also, for each drifter pair *i*, the radial vector is defined as the direction from the eddy center to the paid midpoint, and the squared separation can therefore be expressed as

$$D_{i}{\left(t\right)}^{2}=D_{i,r}^{2}\left(t\right)+D_{i,a}^{2}\left(t\right)$$ (4)

where the subscripts r and a indicate the radial and azimuthal components, respectively. The dispersion for each component is defined as the ensemble mean over all selected drifter pairs. Because the ensemble averaging is applied after the decomposition, the total dispersion $D^{2}$ is not strictly equal to the sum $D_{r}^{2}+D_{a}^{2}$. Here, the resulting discrepancy is less than 1% and the cross-correlation between the radial and azimuthal components is modest (0.3), supporting the robustness and practical validity of the decomposition. We additionally computed along- and cross-SSH-contour dispersion (fig. S3). The results are qualitatively consistent with the radial-azimuthal decomposition. Because the observed eddy remained largely coherent and approximately axisymmetric, the radial-azimuthal decomposition provides a reasonable approximation of along- and cross-eddy transport.

The relative diffusivity is the time derivative of the relative dispersion. Following standard Lagrangian theory (*27*), the directional diffusivity is

$$K_{r}\left(t\right)=\frac{1}{2}\;\frac{dD_{r}^{2}\left(t\right)}{\mathrm{dt}},\;K_{a}\left(t\right)=\frac{1}{2}\;\frac{dD_{a}^{2}\left(t\right)}{\mathrm{dt}}$$ (5)

Here, $K_{r}\left(t\right)$ measures cross-front spreading (inward/outward) spreading, whereas $K_{a}\left(t\right)$ quantifies azimuthal stretching of the flow.

### Four-point group method for calculating vorticity, divergence, and strain rate

Horizontal flow gradients were estimated using a four-point drifter group method, in which a locally linear velocity field is fitted to the positions and velocities of drifters in each group. For a group of four drifters, the horizontal velocity field is approximated by a first-order Taylor expansion about a reference point (*x*_0_, *y*_0_)

$$\begin{array}{c} u=u_{0}+u_{x}\left(x-x_{0}\right)+u_{y}\left(y-y_{0}\right),\\ v=v_{0}+v_{x}\left(x-x_{0}\right)+v_{y}\left(y-y_{0}\right) \end{array}$$ (6)

where 𝑢 and 𝑣 are the zonal and meridional velocities, and ($u_{x}$, $u_{y}$, $v_{x}$, $v_{y}$) are the components of horizontal velocity gradient tensor

$$\nabla V\left(x_{0},y_{0}\right)=\left[\begin{array}{cc} u_{x} & u_{y}\\ v_{x} & v_{y} \end{array}\right]$$ (7)

The gradient components are obtained by performing a least-squares fit to the observed drifter velocities within each four-point group. From this tensor, the verticity vorticity (ζ), horizontal divergence (δ), and strain rate (*S*) can be computed as

$$\zeta =v_{x}-u_{y},\;\delta =u_{x}+v_{y},\;S=\sqrt{{\left(u_{x}-v_{y}\right)}^{2}+{\left(u_{y}+v_{x}\right)}^{2}}$$ (8)

This approach reduces uncertainty in the estimated velocity gradients and yields more robust estimates of ζ, δ, and *S* (*52*). These quantities can capture submesoscale dynamics, as long as the group size remains within the submesoscale range appropriate for resolving submesoscale variability.

## Supplementary Materials

### This PDF file includes:

Figs. S1 to S8 Supplementary Text References
