# Supplementary material for: Direct observations of submesoscale turbulence driving mesoscale eddy leakage
Source: Sci Adv. 2026 Oct 23;12(43):eaef8067. doi: 10.1126/sciadv.aef8067 (PMC13693687; doi:10.1126/sciadv.aef8067)
Supplement: Supplementary file 1 [file sciadv.aef8067_sm.pdf]

Science Advances
AAAS

Supplementary Materials for

Direct observations of submesoscale turbulence driving mesoscale eddy leakage

Haijin Cao *et al.*

Corresponding author: Zhiyou Jing, jingzhiyou@scsio.ac.cn

Sci. Adv. **12**, eaef8067 (2026)
DOI: 10.1126/sciadv.aef8067

This PDF file includes:

Figs. S1 to S8
Supplementary Text
References

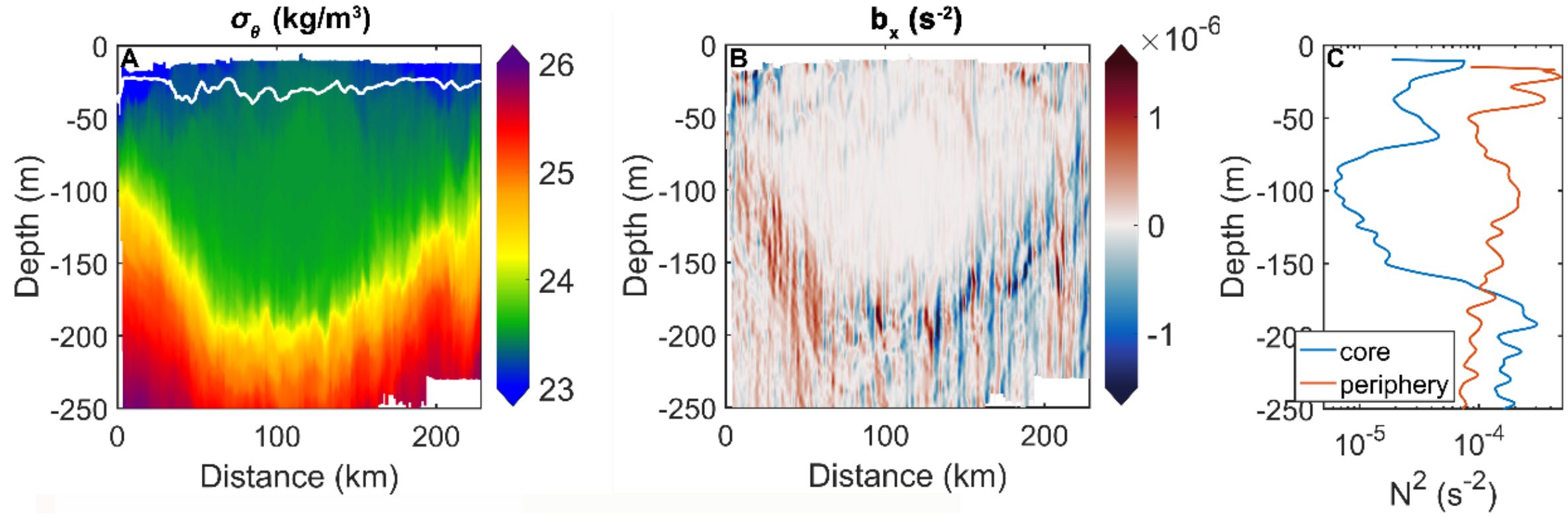

Fig. S1.

Cross-eddy TRIAXUS observations from 12:40 on 9 April to 07:40 on 10 April 2025. (**A**) High-resolution (~500 m) density section for the upper 250 m. The white curve marks the mixed-layer depth, defined using a density threshold of 0.03 kg/m^3 referenced to near-surface density. (**B**) Lateral buoyancy gradient along the transect, $b_x = \partial b/\partial x$, where $b_x = -g(1-\rho/\rho_0)$, g is the gravitational acceleration, ρ is the observed density, and $\rho_0 = 1025$ kg/m^3 is a reference density. The buoyancy gradient varies on kilometer scales, with magnitudes reaching $O(10^{-6})$ s^{-2}, indicative of substantial submesoscale potential energy. (**C**) Brunt–Väisälä frequency profiles for the eddy core and periphery region. A pronounced reduction to 5×10^{-6} s^{-2} in stratification is evident between 75–150 m within the anticyclonic eddy core, associated with isopycnal sinking. This low stratification layer may be favorable for submesoscale instabilities, with small Richardson number.

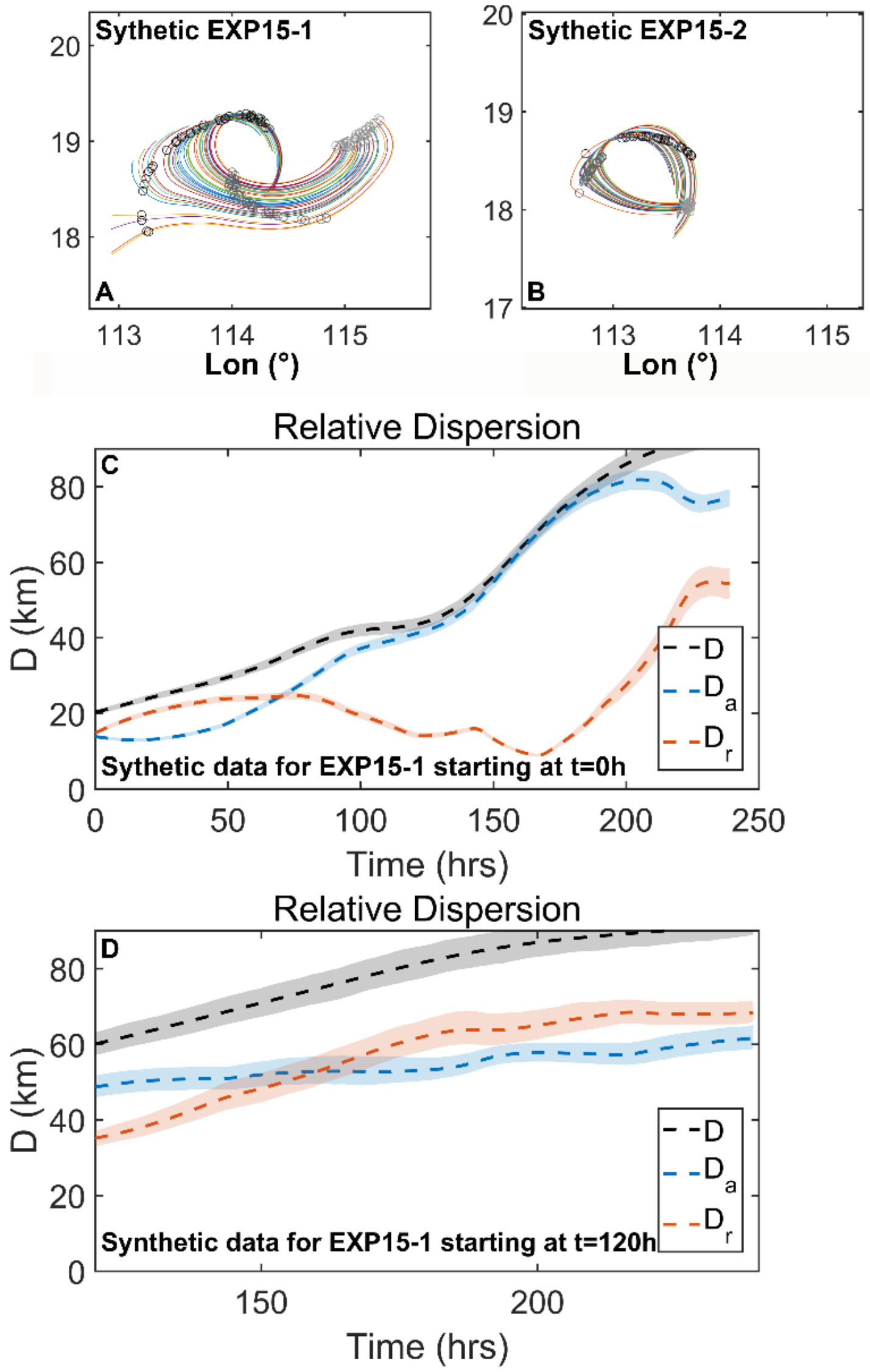

Fig. S2.

Synthetic trajectories based on geostrophic flows. Synthetic drifter trajectories for (**A**) EXP15-1 and (**B**) EXP15-2, computed using geostrophic velocity fields for the early 240 h after deployed. Dots indicate drifter positions at three selected times (t=1h, 96h, and 192h) during the deployment. (**C** and **D**) Comparison of EXP15-1 synthetic trajectories initialized at different deployment times (t =0 h and t = 120 h).

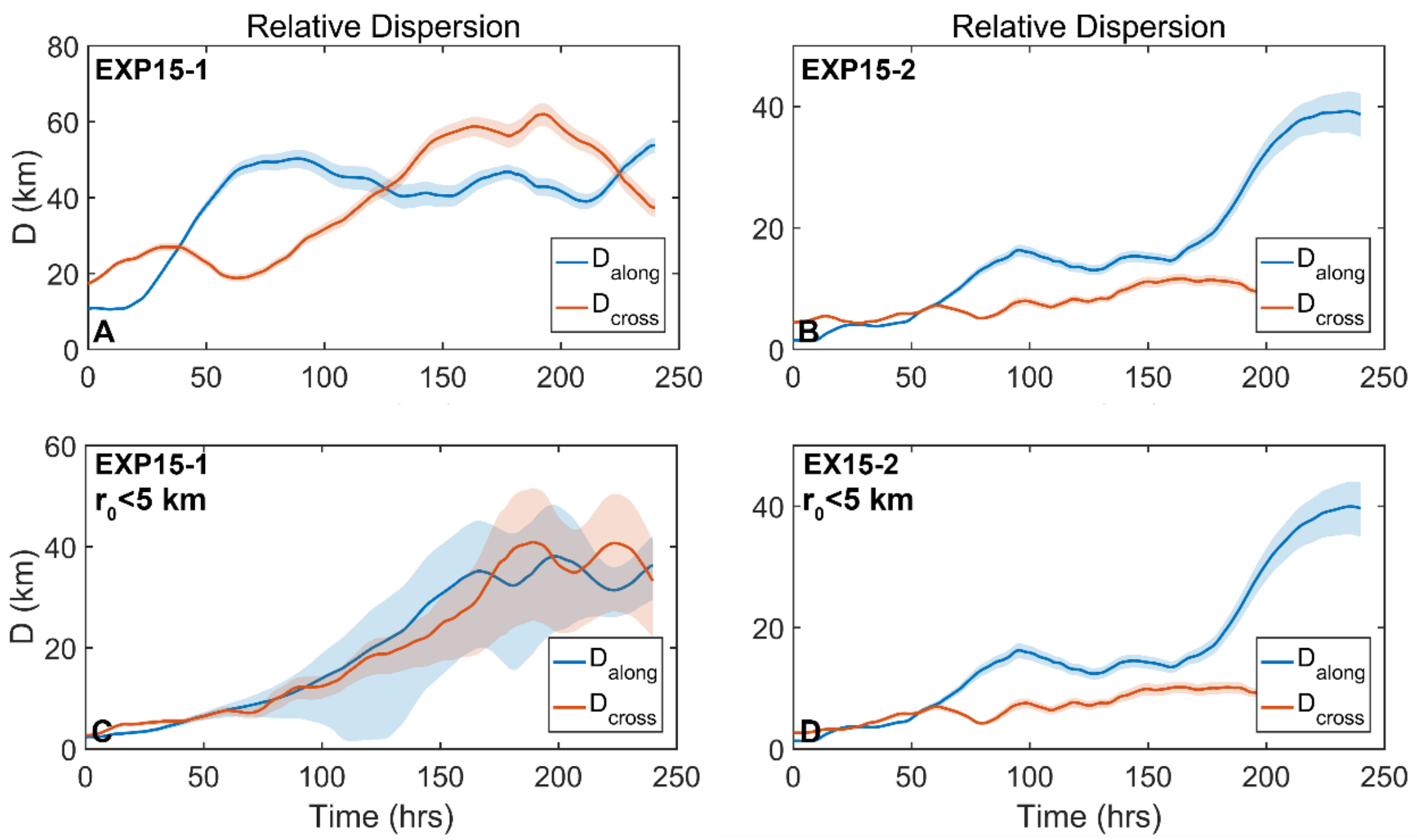

Fig. S3.

Along- and cross-SSH-contour relative dispersion. Relative dispersion projected onto directions parallel (D_{along}) and normal (D_{cross}) to the local SSH contours. Relative dispersion computed using all drifter pairs in (**A**) EXP15-1 and (**B**) EXP15-2. Relative dispersion computed using only drifter pairs with initial separations r_0 < 5 km in (C) EXP15-1 and (D) EXP15-2. The shaded envelopes denote 95% confidence intervals estimated via bootstrapping.

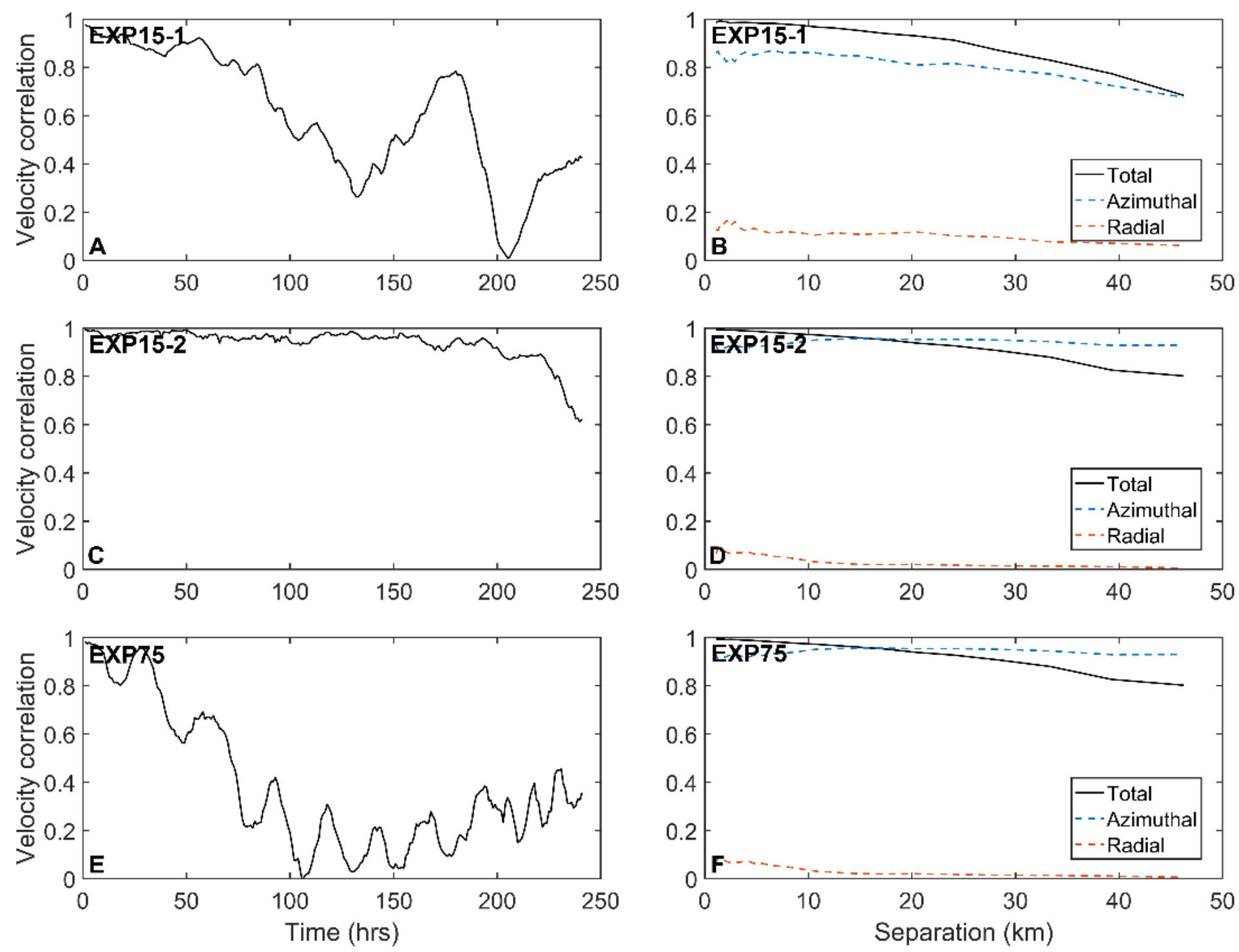

Fig. S4.
Velocity correlation. The velocity correlation is defined as $C = 2\langle \mathbf{v}_i \cdot \mathbf{v}_j \rangle / \left(\langle \mathbf{v}_i^2 \rangle + \langle \mathbf{v}_j^2 \rangle \right)$, where i and j are the drifter index and denote drifter indices and angle brackets indicate ensemble average. Shown are velocity correlations as a function of time for (**A**) EXP15-1, (**B**) EXP15-2, and (**C**) EXP75, and as a function of drifter separation for (**D**) EXP15-1, (**E**) EXP15-2, and (**F**) EXP75. The decomposition into azimuthal and radial components demonstrates that the azimuthal velocity component primarily determines the magnitude of the correlation. A low correlation indicates rapid separation in the radial direction, owing to submesoscale turbulence.

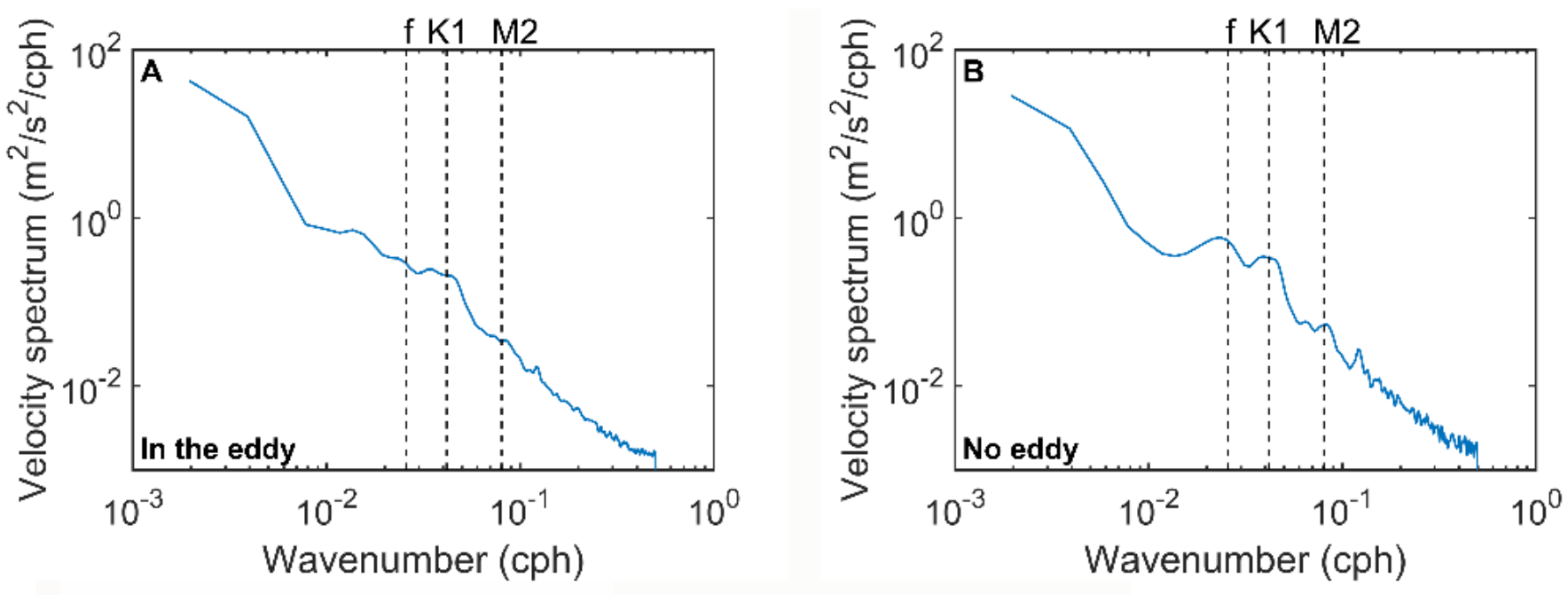

Fig. S5.
Frequency spectra of drifter velocity from EXP15-1. (**A**) Velocity spectrum for April 6–27, when the drifters remained largely within the mesoscale eddy. (**B**) velocity spectrum after June 1, when the eddy had dissipated and the drifters had dispersed. The Coriolis frequency and the K1 and M2 tidal frequencies are marked for reference. In the eddy, near-inertial and tidal signals are not prominent due to energetic submesoscale variability, whereas distinct spectral peaks emerge after the eddy decays.

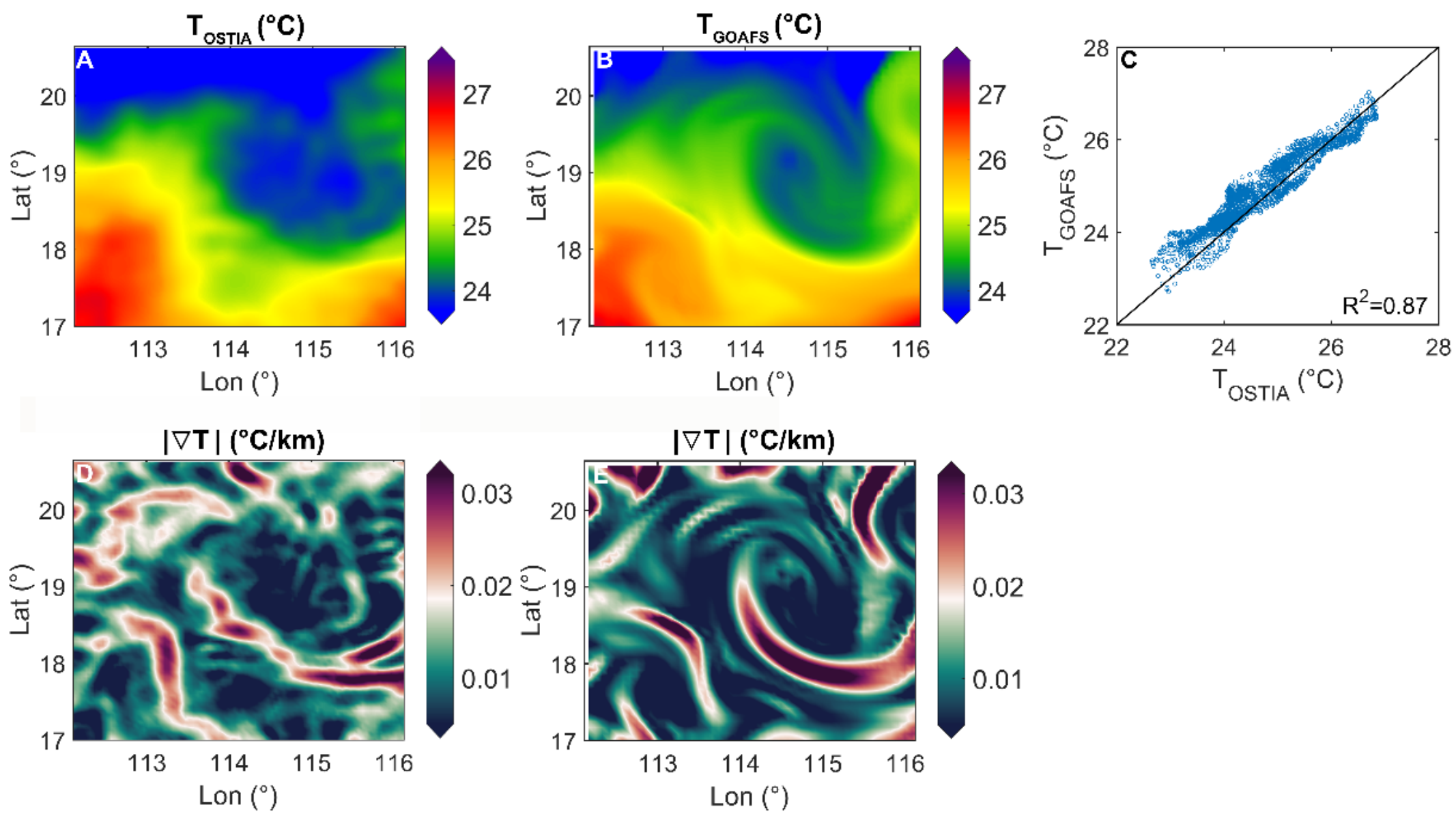

Fig. S6.

Comparison of surface temperature fields and temperature gradient between OSTIA and GOAFS derived from CMEMS. (**A**) Surface temperature on April 6 from the Operational Sea Surface Temperature and Ice Analysis (OSTIA) product. (**B**) Surface temperature on April 6 from the Operational Mercator Global Ocean Analysis and Forecast System (GOAFS). (**C**) Point-to-point comparison of temperature from the two products, with a correlation coefficient of $R^2 = 0.87$. (**D-E**) Corresponding temperature-gradient magnitude fields ($\nabla|T|$). Because GOAFS is a model-based product with data assimilation, it shows smoother and more continuous frontal structures within the eddy region.

Potential influences of wind-driven flows and internal waves

A Lagrangian filtering analysis is employed to separate the velocity components associated with different frequency bands and to directly assess their respective contributions. The observed drifter velocity can be conceptually decomposed as:

$$\mathrm{u}=\mathrm{u}_{\boldsymbol{Ek}}+\mathrm{u}_{\boldsymbol{geo}}+\mathrm{u}_{\boldsymbol{sm}}+\mathrm{u}_{\boldsymbol{NIW}}+\mathrm{u}_{\boldsymbol{tide}}+\mathrm{u}_{\boldsymbol{remaining}} \quad \text{(S1)}$$

where u_{Ek} is the Ekman flow, ugeo is the mesoscale balanced flow, u_{sm} is the submesoscale ageostrophic flow, u_{NIW} represents near-inertial motions, u_{tide} include barotropic and internal tidal motions, and $u_{remaining}$ denotes the residual. In this way, we can evaluate the contributions of the different components to the velocity variance as well as to the convergence/divergence signals.

Ekman flow. As the 15 m drifter drogues were located within the mixed layer during the observations and therefore may be influenced by wind-driven motions. We estimated the wind-driven Ekman velocity using the Global Ocean Hourly Sea Surface Wind and Stress from Scatterometer and Model (CMEMS), which is based on Scatterometer observations for Metop-B and Metop-C (a resolution of 1/8∘). The Ekman velocity was calculated assuming an Ekman layer depth of 30 m. The estimated Ekman flow field is estimated between 0.05-0.1 m/s and spatially uniform over the drifter array. Therefore, although the Ekman flow may contribute to the absolute drifter motion, its contribution to relative dispersion and submesoscale convergence/divergence is expected to be comparatively weak because these quantities depend primarily on spatial velocity differences.

Internal waves including internal tides, near-inertial waves, and internal solitary waves. As in previous SAR observations, the study region is not the hotspot of internal solitary waves, as they are mostly generated by nonlinear steepening of baroclinic tides induced by tide–topography interactions in the Luzon Strait and subsequently propagate northwestward toward the continental shelf (*53*, *54*). To isolate tidal motions and near-inertial oscillations, a band-pass filter of 0.8–1.2f was used to extract near-inertial motions, while band-pass filters centered on the major tidal frequencies (±0.01 cpd bandwidth) were used to isolate tidal signals.

Concerning that wave motions can contribute to submesoscale divergence/convergence, we estimated the divergence/convergence contributions associated with different frequency bands using

$$\delta=\delta_{NIW}+\delta_{tide}+\delta_{LF}+\cdots=\left(\frac{\partial u_{NIW}}{\partial x}+\frac{\partial v_{NIW}}{\partial y}\right)+\left(\frac{\partial u_{tide}}{\partial x}+\frac{\partial v_{tide}}{\partial y}\right)+\left(\frac{\partial u_{LF}}{\partial x}+\frac{\partial v_{LF}}{\partial y}\right)+\cdots \text{(S2)}$$

where δ_{LF} denotes low frequency component (< 0.7f). Here the divergence/convergence is estimated from four-point drifter groups within submesoscale separation distances. As shown in fig. S7 (data of EXP15-1), the low-frequency component contributes substantially more to the strong convergence/divergence events than the wave-related components. Moreover, while wave motions may induce oscillatory signals, they are generally expected to contribute less efficiently to net tracer transport and irreversible relative dispersion than submesoscale turbulence. As also shown in the vorticity field, the low-pass filtering reduces the ageostrophic features but

ageostrophic motions remain clear with $|\zeta/f|>1$ (fig. S8).

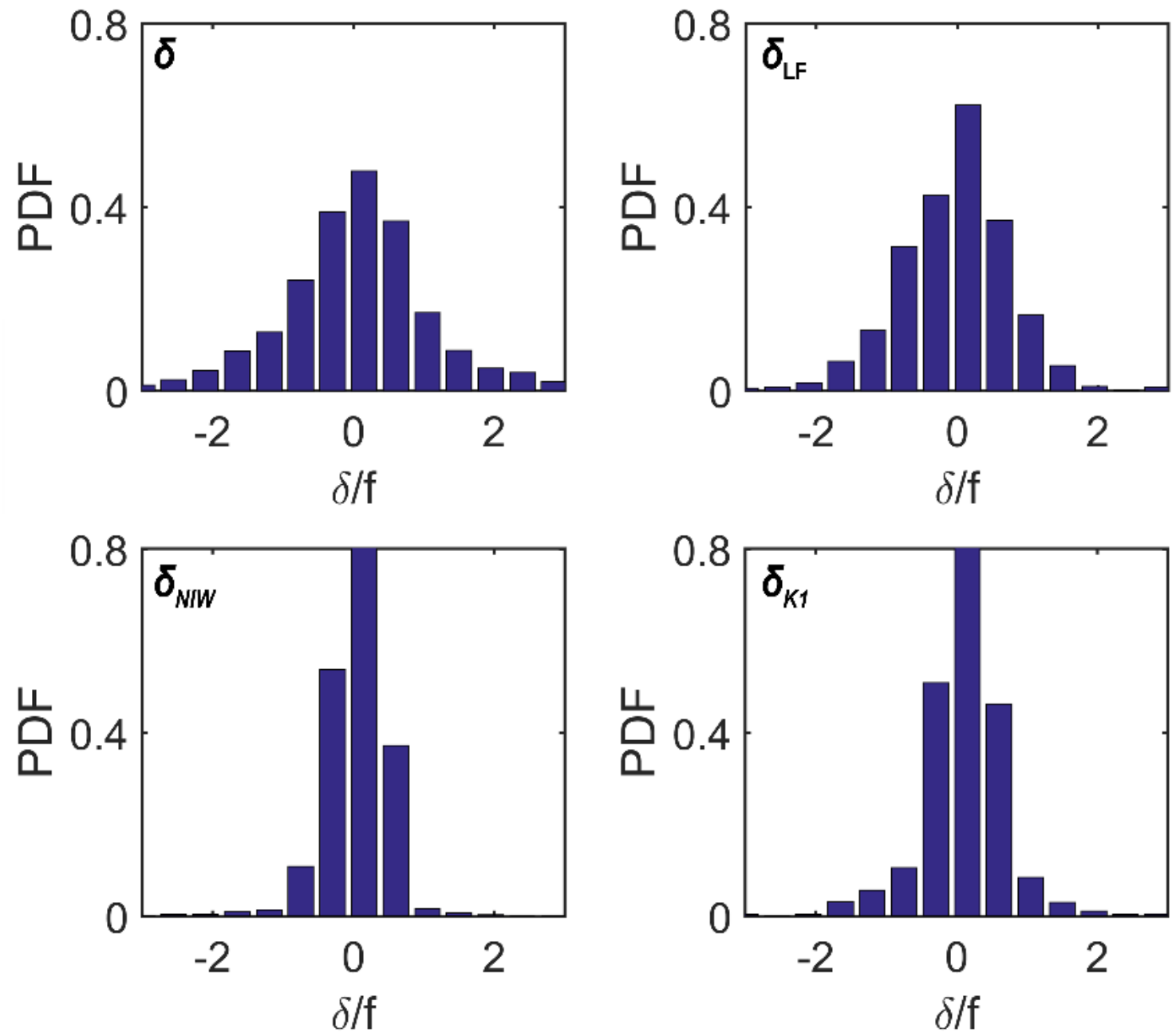

Fig. S7.

Submesoscale divergence decomposed into different components. δ_{LF} denotes low frequency component (< 0.7f). NIW is the near inertial waves. K1 is the diurnal tide.

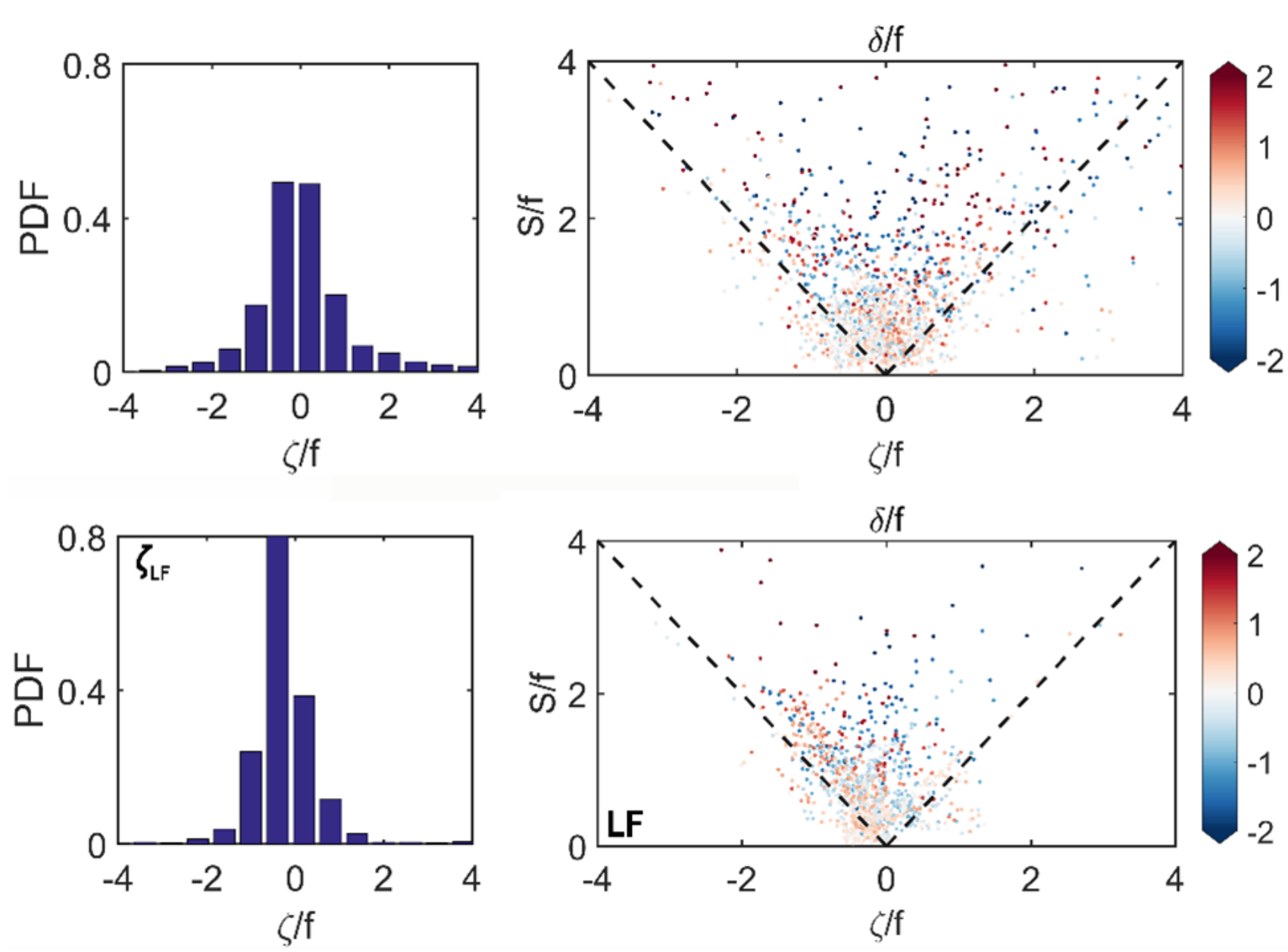

Fig. S8.

Comparison of vorticity derived from original velocities and low-pass velocities.

REFERENCES

1. K. Bryan, Poleward heat transport in the ocean. *Tellus B* **43**, 104–115 (2022).

2. S. R. Jayne, J. Marotzke, The oceanic eddy heat transport. *J. Phys. Oceanogr.* **32**, 3328–3345 (2002).

3. D. B. Chelton, P. Gaube, M. G. Schlax, J. J. Early, R. M. Samelson, The influence of nonlinear mesoscale eddies on near-surface oceanic chlorophyll. *Science* **334**, 328–332 (2011).

4. D. J. McGillicuddy Jr., Mechanisms of physical–biological–biogeochemical interaction at the oceanic mesoscale. *Ann. Rev. Mar. Sci.* **8**, 125–159 (2016).

5. P. B. Rhines, "Mesoscale eddies" in *Encyclopedia of Ocean Sciences*, J. H. Steele, K. K. Turekian, S. A. Thorpe, Eds. (Academic Press, ed. 3, 2019), pp. 115–127.

6. Z. Zhang, W. Wang, B. Qiu, Oceanic mass transport by mesoscale eddies. *Science* **345**, 322–324 (2014).

7. G. Haller, Lagrangian coherent structures. *Annu. Rev. Fluid Mech.* **47**, 137–162 (2015).

8. N. Tarshish, R. Abernathey, C. Zhang, C. O. Dufour, I. Frenger, S. M. Griffies, Identifying Lagrangian coherent vortices in a mesoscale ocean model. *Ocean Model.* **130**, 15–28 (2018).

9. T. Liu, R. Abernathey, A. Sinha, D. Chen, Quantifying Eulerian eddy leakiness in an idealized model. *J. Geophys. Res. Oceans* **124**, 8869–8886 (2019).

10. A. Sanchez-Rios, R. K. Shearman, J. Klymak, E. D'Asaro, C. Lee, Observations of cross-frontal exchange associated with submesoscale features along the North Wall of the Gulf Stream. *Deep-Sea Res. I Oceanogr. Res. Pap.* **163**, 103342 (2020).

11. M. A. Freilich, A. Mahadevan, Coherent pathways for subduction from the surface mixed layer at ocean fronts. *J. Geophys. Res. Oceans* **126**, e2020JC017042 (2021).

12. H. Cao, M. A. Freilich, X. Song, Z. Jing, B. Fox-Kemper, B. Qiu, Isopycnal submesoscale stirring crucially sustaining subsurface chlorophyll maximum in ocean cyclonic eddies. *Geophys. Res. Lett.* **51**, e2023GL105793 (2024).

13. M. M. Omand, E. A. D'Asaro, C. M. Lee, M. J. Perry, N. Briggs, I. Cetinić, A. Mahadevan, Eddy-driven subduction exports particulate organic carbon from the spring bloom. *Science* **348**, 222–225 (2015).

14. A. Mahadevan, The impact of submesoscale physics on primary productivity of plankton. *Ann. Rev. Mar. Sci.* **8**, 161–184 (2016).

15. M. Lévy, P. J. S. Franks, K. S. Smith, The role of submesoscale currents in structuring marine ecosystems. *Nat. Commun.* **9**, 4758 (2018).

16. E. A. D'Asaro, A. Y. Shcherbina, J. M. Klymak, J. Molemaker, G. Novelli, C. M. Guigand, A. C. Haza, B. K. Haus, E. H. Ryan, G. A. Jacobs, H. S. Huntley, N. J. M. Laxague, S. Chen, F. Judt, J. C. McWilliams, R. Barkan, A. D. Kirwan Jr., A. C. Poje, T. M. Özgökmen, Ocean convergence and the dispersion of flotsam. *Proc. Natl. Acad. Sci. U.S.A.* **115**, 1162–1167 (2018).

17. J. C. McWilliams, Submesoscale currents in the ocean. *Proc. R. Soc. A* **472**, 20160117 (2016).

18. J. T. Allen, *Cruise Report: RSS Discovery Cruise 381*, *14 Sep.–03 Oct. 2012: Ocean Surface Mixing* (National Oceanography Centre, 2013).

19. A. Y. Shcherbina, M. A. Sundermeyer, E. Kunze, E. D'Asaro, G. Badin, D. Birch, The LatMix summer campaign: Submesoscale stirring in the upper ocean. *Bull. Am. Meteorol. Soc.* **96**, 1257–1279 (2015).

20. E. A. D'Asaro, D. F. Carlson, M. Chamecki, R. R. Harcourt, B. K. Haus, B. Fox-Kemper, M. J. Molemaker, A. C. Poje, D. Yang, Advances in observing and understanding small-scale open-ocean circulation during the Gulf of Mexico Research Initiative era. *Front. Mar. Sci.* **7**, 355 (2020).

21. Z. Zhang, X. Zhang, B. Qiu, W. Zhao, C. Zhou, X. Huang, Submesoscale currents in the subtropical upper ocean observed by two-year-long high-resolution mooring arrays. *J. Phys. Oceanogr.* **51**, 187–206 (2021).

22. A. Mahadevan, A. Pascual, D. L. Rudnick, S. Ruiz, J. Tintoré, E. D'Asaro, CALYPSO: Observing coherent pathways from the surface ocean to the interior. *Bull. Am. Meteorol. Soc.* **102**, 532–540 (2023).

23. J. T. Farrar, L. Rainville, A. J. Plueddemann, J. M. Klymak, C. M. Lee, M. H. Alford, The sub-mesoscale ocean dynamics experiment (S-MODE). *Bull. Am. Meteorol. Soc.* **106**, E657–E677 (2025).

24. L.-L. Fu, T. Pavelsky, J.-F. Cretaux, R. Morrow, J. T. Farrar, P. Vaze, The surface water and ocean topography mission. *Geophys. Res. Lett.* **51**, e2023GL107652 (2024).

25. M. Archer, J. Wang, P. Klein, C. B. Rocha, H. Sasaki, B. Qiu, Wide-swath satellite altimetry unveils global submesoscale ocean dynamics. *Nature* **640**, 691–696 (2025).

26. B. Qiu, S. Chen, Fine-scale upper-ocean variability in the Kuroshio Extension region from wide-swath SWOT measurements. *J. Phys. Oceanogr.* **55**, 2229–2242 (2025).

27. J. H. LaCasce, Statistics from Lagrangian observations. *Prog. Oceanogr.* **77**, 1–29 (2008).

28. A. C. Poje, T. M. Özgökmen, B. L. Lipphardt Jr., B. K. Haus, E. H. Ryan, A. C. Haza, G. A. Jacobs, A. J. H. M. Reniers, M. J. Olascoaga, G. Novelli, A. Griffa, F. J. Beron-Vera, S. S. Chen, E. Coelho, P. J. Hogan, A. D. Kirwan Jr., H. S. Huntley, A. J. Mariano, Submesoscale dispersion in the vicinity of the Deepwater Horizon spill. *Proc. Natl. Acad. Sci. U.S.A.* **111**, 12693–12698 (2014).

29. S. Essink, V. Hormann, L. R. Centurioni, A. Mahadevan, Can we detect submesoscale motions in drifter pair dispersion? *J. Phys. Oceanogr.* **49**, 2237–2254 (2019).

30. G. Haller, Lagrangian structures and the rate of strain in a partition of two-dimensional turbulence. *Phys. Fluids* **13**, 3365–3385 (2001).

31. F. J. Beron-Vera, M. J. Olascoaga, G. J. Goni, Oceanic mesoscale eddies as revealed by Lagrangian coherent structures. *Geophys. Res. Lett.* **35**, L12603 (2008).

32. Y. K. Qian, J. H. LaCasce, S. Peng, Inferring submesoscale energy spectra in the Gulf of Mexico from surface drifters. *J. Phys. Oceanogr.* **55**, 1475–1491 (2025).

33. P. E. Isachsen, I. Koszalka, J. H. LaCasce, Observed and modeled surface eddy heat fluxes in the eastern Nordic Seas. *J. Geophys. Res.* **117**, C08020 (2012).

34. S. T. Cole, C. Wortham, E. Kunze, W. B. Owens, Eddy stirring and horizontal diffusivity from Argo float observations. *Geophys. Res. Lett.* **42**, 3989–3997 (2015).

35. H. Cao, B. Fox-Kemper, X. Song, Y. Yuan, Z. Jing, Submesoscale stirring drives lateral dispersion and vertical exchange in a cyclonic mesoscale eddy. *J. Geophys. Res. Oceans* **131**, e2025JC023066 (2026).

36. R. Molinari, A. D. Kirwan, Calculations of differential kinematic properties from Lagrangian observations in the western Caribbean Sea. *J. Phys. Oceanogr.* **5**, 483–491 (1975).

37. A. Okubo, C. C. Ebbesmeyer, Determination of vorticity, divergence, and deformation rates from drogue observations. *Deep Sea Res.* **23**, 349–352 (1976).

38. D. Balwada, Q. Xiao, S. Smith, R. Abernathey, A. R. Gray, Vertical fluxes conditioned on vorticity and strain reveal submesoscale ventilation. *J. Phys. Oceanogr.* **51**, 2883–2901 (2021).

39. J. Pearson, B. Fox-Kemper, B. Pearson, H. Chang, B. K. Haus, J. Horstmann, H. S. Huntley, A. D. Kirwan Jr., B. Lund, A. Poje, Biases in structure functions from observations of submesoscale flows. *J. Geophys. Res. Oceans* **125**, e2019JC015769 (2020).

40. J. Dong, B. Fox-Kemper, J. O. Wenegrat, Submesoscales are a significant turbulence source in the global ocean surface boundary layer. *Nat. Commun.* **15**, 9566 (2024).

41. J. Callies, R. Ferrari, J. M. Klymak, J. Gula, Seasonality in submesoscale turbulence. *Nat. Commun.* **6**, 686 (2015).

42. H. Cao, B. Fox-Kemper, Z. Jing, X. Song, Y. Liu, Towards the upper-ocean unbalanced submesoscale motions in the Oleander observations. *J. Phys. Oceanogr.* **53**, 1123–1138 (2023).

43. M. A. Freilich, C. Poirier, M. Dever, E. Alou-Font, J. Allen, A. Cabornero, 3D intrusions transport active surface microbial assemblages to the dark ocean. *Proc. Natl. Acad. Sci. U.S.A.* **121**, e2319937121 (2024).

44. E. Kunze, M. A. Sundermeyer, The role of intermittency in internal-wave shear dispersion. *J. Phys. Oceanogr.* **45**, 2979–2990 (2015).

45. L. N. Thomas, A. Tandon, A. Mahadevan, "Submesoscale processes and dynamics" in *Ocean Modeling in an Eddying Regime*, M. W. Hecht, H. Hasumi, Eds. (American Geophysical Union, 2008), pp. 17–38.

46. J. C. McWilliams, Submesoscale surface fronts and filaments: Secondary circulation, buoyancy flux, and frontogenesis. *J. Fluid Mech.* **823**, 391–432 (2017).

47. H. T. Pham, S. Sarkar, Ageostrophic secondary circulation at a submesoscale front and the formation of gravity currents. *J. Phys. Oceanogr.* **48**, 2507–2529 (2018).

48. K. Srinivasan, R. Barkan, J. C. McWilliams, A forward energy flux at submesoscales driven by frontogenesis. *J. Phys. Oceanogr.* **53**, 287–305 (2023).

49. Z. Zhang, B. Qiu, Evolution of submesoscale ageostrophic motions through the life cycle of oceanic mesoscale eddies. *Geophys. Res. Lett.* **45**, 11847–11855 (2018).

50. Z. Zhang, B. Qiu, Surface chlorophyll enhancement in mesoscale eddies by submesoscale spiral bands. *Geophys. Res. Lett.* **47**, e2020GL088820 (2020).

51. R. Lumpkin, T. Özgökmen, L. Centurioni, Advances in the application of surface drifters. *Ann. Rev. Mar. Sci.* **9**, 59–81 (2017).

52. A. Shcherbina, E. A. D'Asaro, C. M. Lee, J. M. Klymak, Statistics of vertical vorticity, divergence, and strain in a developed submesoscale turbulence field. *Geophys. Res. Lett.* **40**, 4706–4711 (2013).

53. Z. Zhao, V. Klemas, Q. Zheng, X. H. Yan, Remote sensing evidence for baroclinic tide origin of internal solitary waves in the northeastern South China Sea. *Geophys. Res. Lett.* **31**, L06302 (2004).

54. Z. Zhao, M. H. Alford, Source and propagation of internal solitary waves in the northeastern South China Sea, *J. Geophys. Res.* **111**, C11012 (2006).
